# Pin1At regulates PIN1 polar localization and root gravitropism

**DOI:** 10.1038/ncomms10430

**Published:** 2016-01-21

**Authors:** Wanyan Xi, Ximing Gong, Qiaoyun Yang, Hao Yu, Yih-Cherng Liou

**Affiliations:** 1 Department of Biological Sciences, Faculty of Science, National University of Singapore, Lower Kent Ridge Road, 10 Science Drive 4, Singapore 117543, Singapore; 2 Temasek Life Sciences Laboratory, National University of Singapore, 1 Research Link, Singapore 117604, Singapore; 3 Graduate School for Integrative Sciences and Engineering, National University of Singapore, Singapore 117573, Singapore

## Abstract

Root gravitropism allows plants to establish root systems and its regulation depends on polar auxin transport mediated by PIN-FORMED (PIN) auxin transporters. PINOID (PID) and PROTEIN PHOSPHATASE 2A (PP2A) act antagonistically on reversible phosphorylation of PINs. This regulates polar PIN distribution and auxin transport. Here we show that a peptidyl-prolyl *cis/trans* isomerase Pin1At regulates root gravitropism. Downregulation of *Pin1At* suppresses root agravitropic phenotypes of *pp2aa* and *35S:PID*, while overexpression of *Pin1At* affects root gravitropic responses and enhances the *pp2aa* agravitropic phenotype. Pin1At also affects auxin transport and polar localization of PIN1 in stele cells, which is mediated by PID and PP2A. Furthermore, Pin1At catalyses the conformational change of the phosphorylated Ser/Thr-Pro motifs of PIN1. Thus, Pin1At mediates the conformational dynamics of PIN1 and affects PID- and PP2A-mediated regulation of PIN1 polar localization, which correlates with the regulation of root gravitropism.

## Abstract (web summary)

Asymmetric transport of auxin allows plants to orient growth of their roots towards gravity. Here, the authors show that the peptidyl-prolyl *cis/trans* isomerase Pin1At contributes to root gravitropism and propose that it acts by altering the polarity of the PIN1 auxin transport protein.

## Graphical abstract

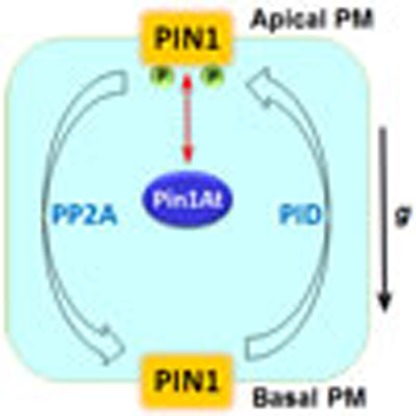

Plant organs are able to sense gravity to grow in the appropriate direction, a response called gravitropism. Root gravitropism allows roots to grow downward into the soil for better absorption of nutrients and water, which is governed by complex physiological and molecular processes^1^,^2^. In general, the gravitropic response pathway comprises three phases: gravity perception, signal transduction and asymmetric growth response^3^,^4^,^5^. These sequential steps involve coordinated action of many molecules, among which the phytohormone auxin is regarded as a master regulator. On gravistimulation, an auxin gradient is formed between the upper and lower sides of the root, which then drives root bending^6^.

Auxin moves in a cell-to-cell manner and is distributed asymmetrically throughout the plant body. In *Arabidopsis*, polar transport of auxin is mainly mediated by three families of plasma membrane (PM)-associated transporter proteins, including AUXIN RESISTANT1/LIKE AUX1 influx carriers^7^,^8^,^9^, PIN-FORMED (PIN) efflux carriers^10^ and P-GLYCOPROTEIN ATP-binding cassette transporters^11^,^12^. Among these transporters, PIN auxin efflux carriers play pivotal roles in directing intercellular auxin flow and mediating the auxin-regulated developmental processes through their asymmetric and polar localization at the PM^13^. Polar PIN targeting is phosphorylation dependent, which is mediated by antagonistic effects of the protein serine/threonine (Ser/Thr) kinase PINOID (PID) and the phosphatase PROTEIN PHOSPHATASE 2A (PP2A)^14^. Both overexpression of *PID* and reduced *PP2A* activity increase PIN phosphorylation, resulting in a basal-to-apical shift in PIN polarity and consequently a root agravitropic phenotype^14^,^15^. Sequence and functional analyses have identified several conserved phosphorylation sites in the large central PIN hydrophilic loop, including three TPRXS(N/S) motifs and one Ser337/Thr340 site^16^,^17^. Reversible phosphorylation of those residues determines the polar PIN targeting and auxin-mediated plant development.

Human PIN1-type peptidyl-prolyl *cis*/*trans* isomerases (PPIases) have emerged as critical regulators of signal transduction pathways in various organisms^18^. This type of isomerases specifically recognize phosphorylated Ser/Thr residues preceding Pro (pSer/Thr-Pro) and catalyse *cis/trans* conformational change in a subset of phosphoproteins, thus altering relevant protein properties including stability, subcellular localization, phosphorylation status, catalytic activity and protein–protein interaction^19^,^20^,^21^. Although studies in metazoans have advanced our understanding of PIN1-type PPIases, the biological roles of their orthologues in plants are still largely unknown. In a previous study, we have shown that the only *Arabidopsis* orthologue of PIN1-type PPIases, Pin1At, regulates flowering time via phosphorylation-dependent isomerization of two MADS-box transcription factors^22^. In addition, another *Arabidopsis* PPIase, AtFKBP42 or TWISTED DWARF 1 that belongs to the subfamily of FKBP (FK506-binding protein)-type immunophilins, has been suggested to interact with PID, to affect auxin efflux mediated by an auxin exporter, ABC transporter B1 (refs 23, 24). Very recently, LATERAL ROOTLESS2, a cyclophilin-type PPIase, has been found to promote AUXIN/INDOLE-3-ACETIC ACID (Aux/IAA) protein degradation to regulate auxin signalling in rice^25^.

Here we present evidence that in *Arabidopsis* Pin1At plays a hitherto unknown role in controlling root gravitropism and modulating the polarity of the efflux carrier PIN1 and auxin transport mediated by PID and PP2A. In addition, we show that Pin1At accelerates the *cis/trans* conformational change of phosphorylated Ser/Thr-Pro motifs in the central PIN1 hydrophilic loop. Our findings suggest that Pin1At catalyses the conformational dynamics of PIN1 and mediates the antagonistic effects of PID and PP2A on PIN1 polar localization, which correlates to regulation of root gravitropism.

## Results

### Alteration in *Pin1At* expression affects root gravitropism

**Figure 1:**
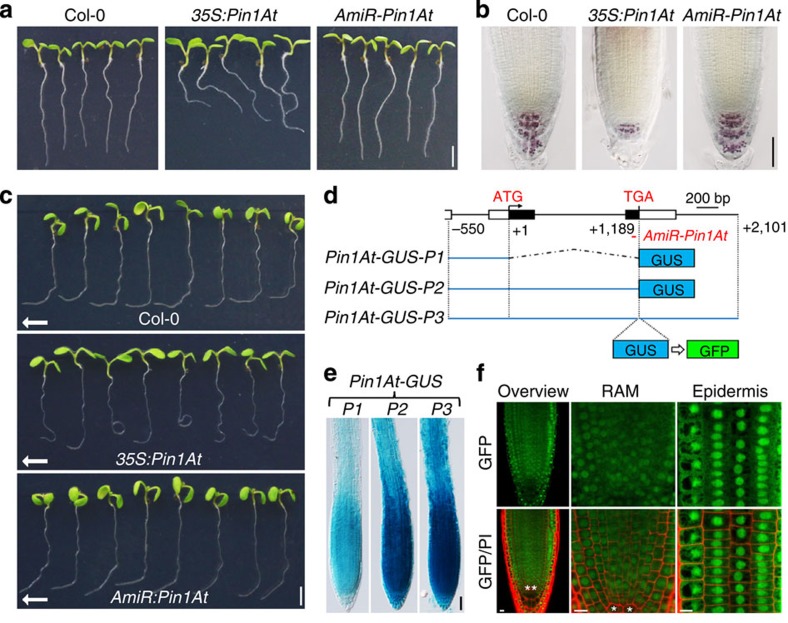
***Pin1At*** **mediates root gravitropic responses.** (**a**) Root phenotype of wild-type Col-0, *35S:Pin1At* and *AmiR-Pin1At* plants at 4 DAG. DAG, days after germination. Scale bar, 2.5 mm. (**b**) Lugol's staining of root tips of wild-type Col-0, *35S:Pin1At* and *AmiR-Pin1At* seedlings. Scale bar, 50 μm. (**c**) Comparison of root gravity responses of wild-type Col-0, *35S:Pin1At* and *AmiR-Pin1At* seedlings after gravi-stimulation. Arrows indicate the new gravity vector. Scale bar, 2.5 mm. (**d**) Schematic diagram of *Pin1At-GUS-P1*, *P2* and *P3* constructs. The target site of the AmiR in *AmiR-Pin1At* is indicated. Exons in the coding region are indicated by black boxes, whereas untranslated regions are indicated by white boxes. Part of the 3′-untranslated region of the gene preceding *Pin1At* is also included in these three constructs. *pPin1At:Pin1At-GFP* was constructed by replacing *GUS* with *GFP* in *Pin1At-GUS-P3*. (**e**) GUS staining of primary roots of representative *Pin1At-GUS-P1*, *P2* and *P3* transgenic plants. Scale bar, 50 μm. (**f**) Visualization of Pin1At-GFP localization in primary roots of *pPin1At:Pin1At-GFP* by confocal laser scanning microscopy (CLSM). **Quiescent centre cells. PI, propidium iodide; RAM, root apical meristem. Scale bars, 10 μm.

In the course of investigating the biological function of *Pin1At*^22^, we found that transgenic plants overexpressing *Pin1At* under the control of 35S promoter (*35S:Pin1At*) displayed an obvious agravitropic phenotype and slightly reduced root growth when they were germinated and grown on Murashige and Skoog (MS) agar plates oriented vertically for 4 days (Fig. 1a and Supplementary Fig. 1a,b). As plants sense gravity stimulus via columella root cap cells that contain starch-filled plastids called amyloplasts, we stained root tips with Lugol's solution and observed a reduced number of columella cells containing stained starch granules in *35S:Pin1At* than in wild-type plants (Fig. 1b and Supplementary Fig. 2). This observation indicates that reduced gravity response exhibited by *35S:Pin1At* might be attributable to a decreased population of columella cells.

To further understand the function of *Pin1At* in the control of root gravitropism, we created *Pin1At* knockdown plants (*AmiR-Pin1At*) by artificial microRNA (AmiR) interference^26^, as insertion mutants of *Pin1At* were unavailable in public resources. The AmiR was designed to specifically target the 3′-region of the *Pin1At* transcript (Fig. 1d). We created 17 *AmiR-Pin1At* lines, in which *Pin1At* expression was downregulated to different extents (Supplementary Fig. 3a). These lines exhibited similar root phenotypes and the strongest line (number 15) with the lowest expression of *Pin1At* was used for further investigations. Although *AmiR-Pin1At* plants displayed normal root gravitropic growth on MS agar plates oriented vertically for 4 days and a similar Lugol staining pattern to that of wild-type plants (Fig. 1a,b), they were defective in gravitropic responses and their roots grew faster (Fig. 1c and Supplementary Fig. 1b,c). We reoriented the seedlings by 90° relative to the gravity vector and compared the angles of root curvature at 24 h after reorientation (Fig. 1c and Supplementary Fig. 1c). The angles of root gravitropic bending for wild-type plants were around 90°, whereas *AmiR-Pin1At* plants exhibited obvious decreases in bending with the angles ranging from 50° to 70°. Root gravitropic defects were much stronger in *35S:Pin1At* plants, many of which had bending angles >90° and tended to form irregular loops. We also performed a time-course study of root gravitropic responses and found that the gravitropic response of both *35S:Pin1At* and *AmiR-Pin1At* was slower than that of wild-type plants (Supplementary Fig. 1d). The root gravitropic phenotype when oriented vertically and the defects in gravitropic responses were similarly observed in other independent *35S:Pin1At* and *AmiR-Pin1At* lines (Supplementary Fig. 1e,f). These results, together with the observation of downregulation and upregulation of *Pin1At* in the roots of *AmiR-Pin1At* and *35S:Pin1At* (Supplementary Fig. 3b), respectively, suggest that *Pin1At* plays an important role in the control of root gravitropism.

### *Pin1At* is highly expressed in roots

To monitor the detailed expression pattern of *Pin1At* in roots, we generated three *Pin1At:β-glucuronidase* (*GUS*) reporter constructs: *Pin1At-GUS-P1*, *P2* and *P3*. In *Pin1At-GUS-P1*, a 550-bp *Pin1At* promoter sequence was transcriptionally fused with the *GUS* gene, whereas 1.7- and 2.6-kb genomic fragments were translationally fused to *GUS* in *Pin1At-GUS-P2* and *P3*, respectively (Fig. 1d). The staining patterns shown by most of the transgenic lines for each construct were similar and a representative line for each construct was selected for further analysis. These three lines exhibited a similar GUS staining pattern in roots, with the strongest staining all observed in the meristematic zone and elongation zone (Fig. 1e and Supplementary Fig. 4). The overall staining intensity in the roots of *Pin1At-GUS-P2* and *P3* was much stronger than that in *Pin1At-GUS-P1*, implying that the genomic region of *Pin1At* succeeding the start codon might contain *cis*-element(s) required for upregulation of *Pin1At*.

We further created *pPin1At:Pin1At-GFP* through replacing *GUS* with a green fluorescent protein (*GFP*) reporter in *Pin1At-GUS-P3* (Fig. 1d). Consistent with the *Pin1At* expression pattern revealed by GUS staining (Fig. 1e), Pin1At-GFP was also mainly expressed in the meristematic zone and elongation zone (Fig. 1f and Supplementary Fig. 4). The GFP fluorescence was observed in both the cytoplasm and nucleus, which is in agreement with the subcellular localization results revealed in *Arabidopsis* protoplasts^22^. The expression pattern of Pin1At in roots and its effect on root gravitropic response suggest that *Pin1At* contributes to the regulation of root gravitropism.

### The root gravitropic defect of *pp2aa* is affected by *Pin1At*

It has been suggested that human Pin1 regulates the conformational change of its substrate phosphoproteins in concert with other kinases/phosphatases^27^. In *Arabidopsis*, the antagonistic interaction between PID kinase and PP2A phosphatases plays a key role in controlling root gravitropism^28^. In addition, the expression pattern of *Pin1At* in roots (Fig. 1e and Supplementary Fig. 4) was similar to that of *PP2AA1*, one of the three closely related regulatory A subunits of the PP2A complex (*PP2AA1-3*)^14^. These results prompted us to investigate whether regulation of root gravitropism by *Pin1At* is relevant to PID and PP2A. To this end, we first crossed *35S:Pin1At* and *AmiR-Pin1At* with loss-of-function mutants of *PP2AA1*, *PP2AA2* and *PP2AA3*, to examine their genetic effects on root gravitropism.

**Figure 2:**
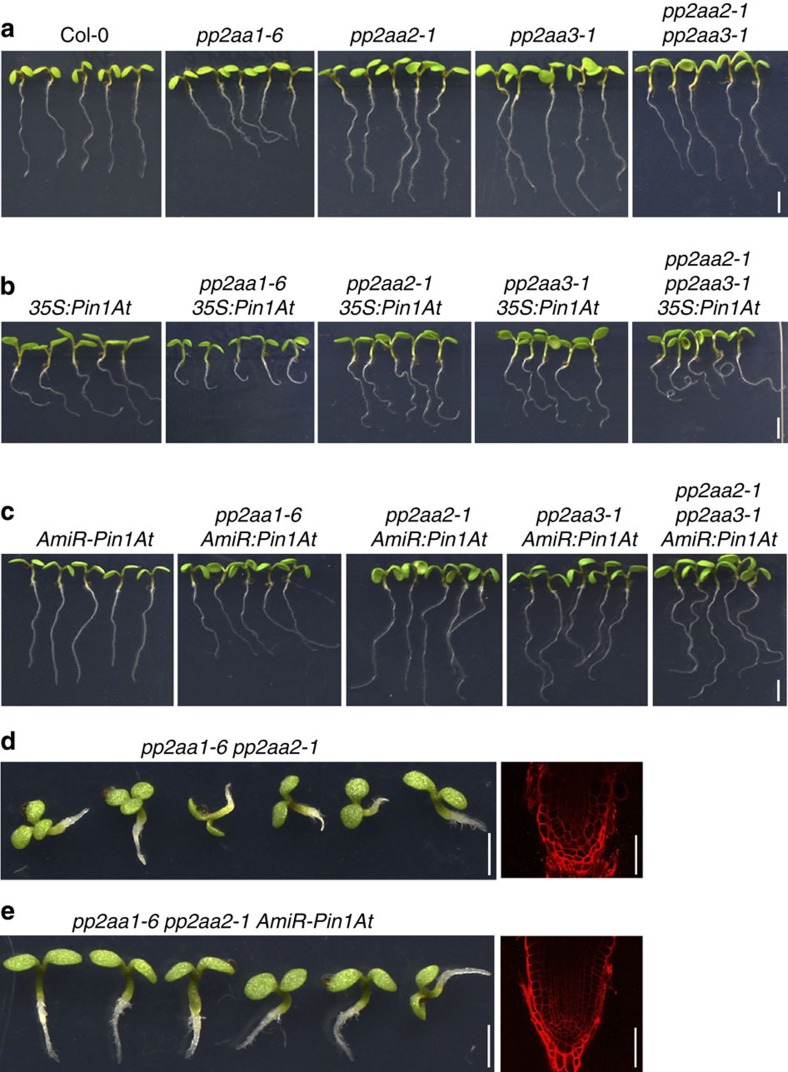
***Pin1At*** **affects the root gravitropic defect in loss-of-function mutants of** ***PP2AAs***. (**a**) Comparison of root gravitropic responses of loss-of-function mutants of *PP2AAs* at 4 DAG. Scale bar, 2.5 mm. (**b**) Comparison of root gravitropic responses of *35S:Pin1At* in various *pp2aa* mutant backgrounds at 4 DAG. Scale bar, 2.5 mm. (**c**) Comparison of root gravitropic responses of *AmiR-Pin1At* in various *pp2aa* mutant backgrounds at 4 DAG. Scale bar, 2.5 mm. (**d**,**e**) Comparison of root gravitropic responses and root meristem organization of *pp2aa1-6 pp2aa2-1* (**d**) and *pp2aa1-6 pp2aa2-1 AmiR-Pin1At* (**e**) at 4 DAG. Left panels show root gravitropic responses (scale bars, 2.5 mm), whereas right panels show the root meristem organization indicated by confocal laser scanning microscopy (CLSM) optical sections of propidium iodide (PI)-stained root tips of representative seedlings (scale bars, 50 μm).

Consistent with previous reports^15^,^29^,^30^,^31^, *pp2aa1-6* single loss-of-function mutants exhibited reduced root length and a defect in gravitropic bending, whereas *pp2aa2-1* and *pp2aa3-1* single mutants showed mostly normal root development (Fig. 2a and Supplementary Fig. 5a). Combination of *pp2aa2-1* and *pp2aa3-1* displayed an observable agravitropic phenotype (Fig. 2a), whereas a combination of *pp2aa1-6* with either *pp2aa2-1* or *pp2aa3-1* resulted in a severer agravitropic phenotype than *pp2aa1-6* alone^14^ (Fig. 2d). These observations suggest that despite the functional redundancy among PP2AA1-3, PP2AA1 plays a dominant role in regulating root development. Overexpression of *Pin1At* in *35S:Pin1At* enhanced the agravitropic phenotype of *pp2aa1-6*, generating coiled roots with significantly reduced length, while its effect on *pp2aa2-1* and *pp2aa3-1* was generally similar to that on wild-type plants (Fig. 2b and Supplementary Fig. 5a). The agravitropic defect of *pp2aa2-1 pp2aa3-1* was also exacerbated by *35S:Pin1At* (Fig. 2b). These results demonstrate a synergistic effect of overexpression of *Pin1At* and loss-of-function of *PP2AAs* on enhancing the root agravitropic phenotype.

In contrast to *35S:Pin1At*, downregulation of *Pin1At* in *AmiR-Pin1At* partially suppressed the root defects of *pp2aa1-6* and *pp2aa2-1 pp2aa3-1*, including root agravitropism and reduced length (Fig. 2c and Supplementary Fig. 5b). Furthermore, *AmiR-Pin1At* greatly suppressed the strong root defects of *pp2aa1-6 pp2aa2-1*, such as root agravitropism and root meristem collapse (Fig. 2d,e). Nearly 71% of the *pp2aa1-6 pp2aa2-1 AmiR-Pin1At* roots (*n*=42) grew towards the gravity vector, whereas only 15% (*n*=45) of the *pp2aa1-6 pp2aa2-1* roots could grow downward. These results suggest that Pin1At and PP2AAs have opposite effects on root gravitropism.

### *AmiR-Pin1At* suppresses root agravitropism of *35S:PID*

**Figure 3:**
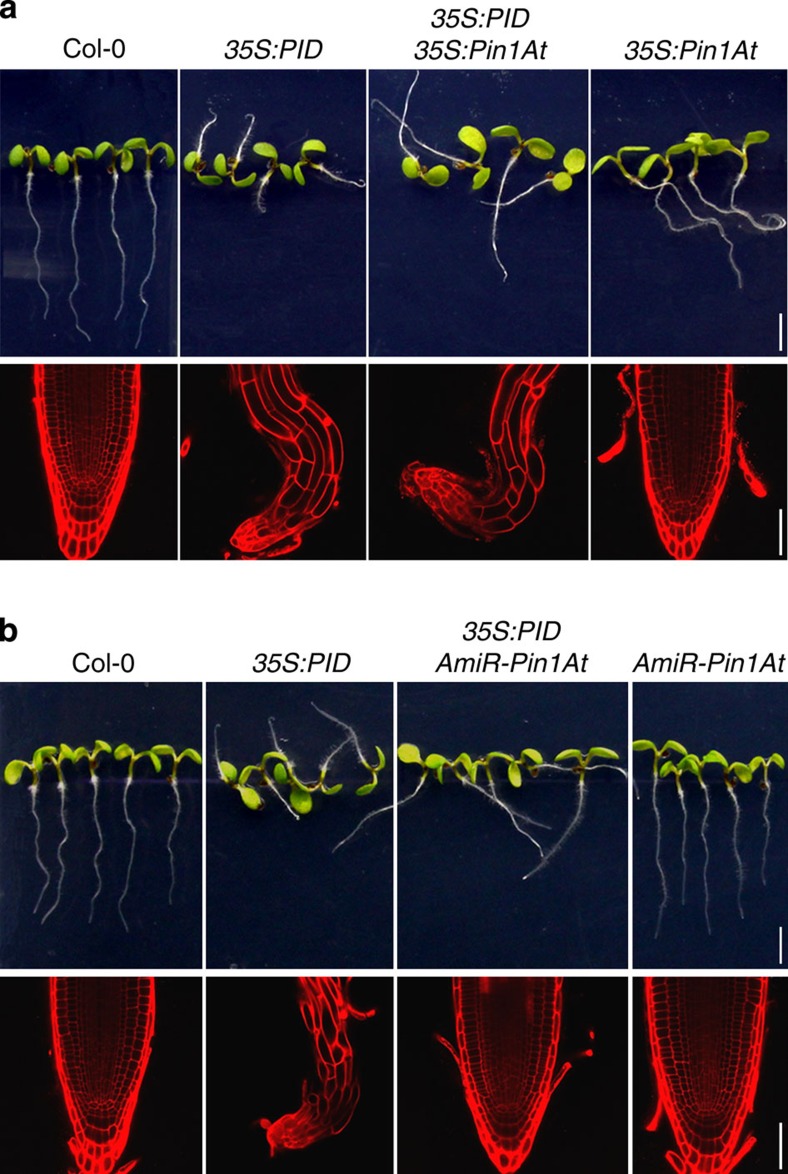
***Pin1At*** **affects the root gravitropic defect in** ***35S:PID***. (**a**) Effect of *35S:Pin1At* on the root gravitropic response and root meristem organization of *35S:PID*. (**b**) Effect of *AmiR:Pin1At* on the root gravitropic response and root meristem organization of *35S:PID*. Upper panels show root gravitropic responses of various seedlings at 4 DAG (scale bars, 2.5 mm), whereas lower panels show the root meristem organization indicated by confocal laser scanning microscopy (CLSM) optical sections of propidium iodide (PI)-stained root tips of representative seedlings (scale bars, 50 μm).

To test the genetic interaction between *Pin1At* and *PID*, we introduced *35S:Pin1At* and *AmiR-Pin1At* into *PID* gain-of-function plants, which exhibited similar root defects, such as root agravitropism and root meristem collapse, to loss-of-function mutants of *PP2AAs*^14^,^32^,^33^. Compared with *35S:PID*, *35S:Pin1At 35S:PID* showed a similarly strong agravitropic root phenotype, but produced much more collapsed primary root meristems and longer roots (Fig. 3a and Supplementary Fig. 6). In contrast, *AmiR-Pin1At* suppressed several strong root defects of *35S:PID*, including root agravitropism, root meristem collapse and reduced length (Fig. 3b and Supplementary Fig. 6). These observations, together with the results from *pp2aa* mutants (Fig. 2), indicate that *Pin1At* could be involved in modulating the antagonistic interaction between PID kinase and PP2A phosphatases to affect root gravitropism.

### Auxin transport is affected by *Pin1At*

**Figure 4:**
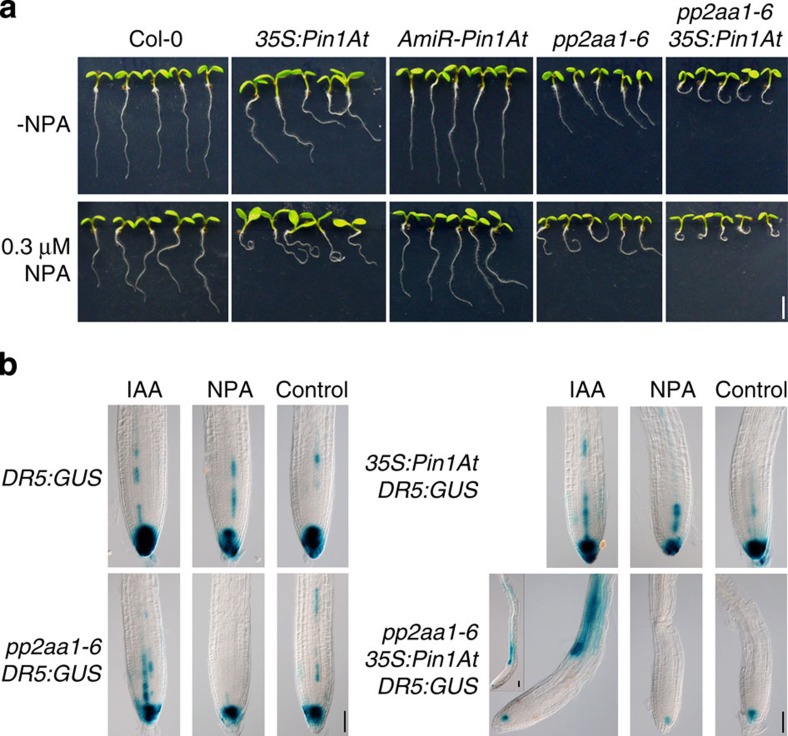
***Pin1At*** **affects auxin transport.** (**a**) NPA effect on root gravitropic responses of wild-type, *35S:Pin1At*, *AmiR-Pin1At*, *pp2aa1-6* and *pp2aa1-6 35S:Pin1At* plants at 4 DAG. Scale bar, 2.5 mm. (**b**) Comparison of the auxin-inducible *DR5:GUS* reporter gene expression in root tips for rootward auxin transport assay. IAA, NPA or MOCK-treated root tips were subjected to GUS staining to visualize rootward auxin transport. Scale bars, 50 μm.

As PID kinase and PP2A phosphatases antagonistically regulate auxin flux that directly controls the root gravitropic response^14^, we then tested whether *Pin1At* also affects auxin transport in seedlings with various genetic backgrounds. We treated seedlings grown vertically with the auxin transport inhibitor 1-*N*-naphthylphthalamic acid (NPA), which blocks tropic curvature responses. Consistent with a previous observation^29^, NPA treatment enhanced the agravitropic phenotype of *pp2aa1-6*. A similar effect was observed for *35S:Pin1At* and *pp2aa1-6 35S:Pin1At*. In contrast, both *AmiR-Pin1At* and wild-type plants displayed a weak response to NPA treatment (Fig. 4a). These results imply that overexpression of *Pin1At* may alter sensitivity to auxin transport inhibitors.

We further analysed changes in auxin distribution using the reporter line (*DR5:GUS*) in which the *GUS* reporter gene is driven by a synthetic auxin-responsive promoter *DR5* (refs 34, 35). Auxin is transported in the root in either a rootward or a shootward pattern. Shoot-derived auxin is transported to the root tip through the central cylinder (stele), whereas auxin is also transported from the root tip towards the root-shoot junction via the outer cell layers^36^. We applied the natural auxin IAA and NPA, to visualize auxin response patterns in the roots of *DR5:GUS* with different genetic backgrounds. In the control treatment for rootward *DR5:GUS* assays, *DR5:GUS* reporter activity was reduced in the root tips of both *pp2aa1-6* and *35S:Pin1At*, and the reduction was even more evident in *pp2aa1-6 35S:Pin1At* (Fig. 4b). Although NPA consistently reduced GUS staining in all genotypes examined, IAA treatment only increased the number of cells with strong GUS staining in wild-type root tips (Fig. 4b), suggesting that rootward IAA transport decreases moderately in *pp2aa1-6* and *35S:Pin1At*, but greatly in *pp2aa1-6 35S:Pin1At*. In contrast, *AmiR-Pin1At* restored dramatically *DR5:GUS* expression in *35S:PID* root tips (Supplementary Fig. 7), further suggesting that *Pin1At* has a negative effect on rootward auxin transport.

In addition, we also examined changes in rootward auxin distribution at high spatial resolution using an established Aux/IAA-based auxin signalling sensor termed DII-VENUS, which could immediately detect dynamic changes in endogenous auxin distribution and responses^37^. IAA treatment promoted DII-VENUS degradation, which was partly suppressed in *35:Pin1At* (Supplementary Fig. 8), further supporting the hypothesis that Pin1At compromises rootward auxin transport.

Similarly, shootward *DR5:GUS* assays showed that GUS staining intensity was weaker in the roots of *pp2aa1-6* and *35S:Pin1At* than in those of wild-type plants. The staining was greatly diminished in *pp2aa1-6 35S:Pin1At* roots (Supplementary Fig. 9). These observations suggest that *Pin1At* negatively regulates both rootward and shootward auxin transport.

### *Pin1At* affects subcellular localization of PIN1

**Figure 5:**
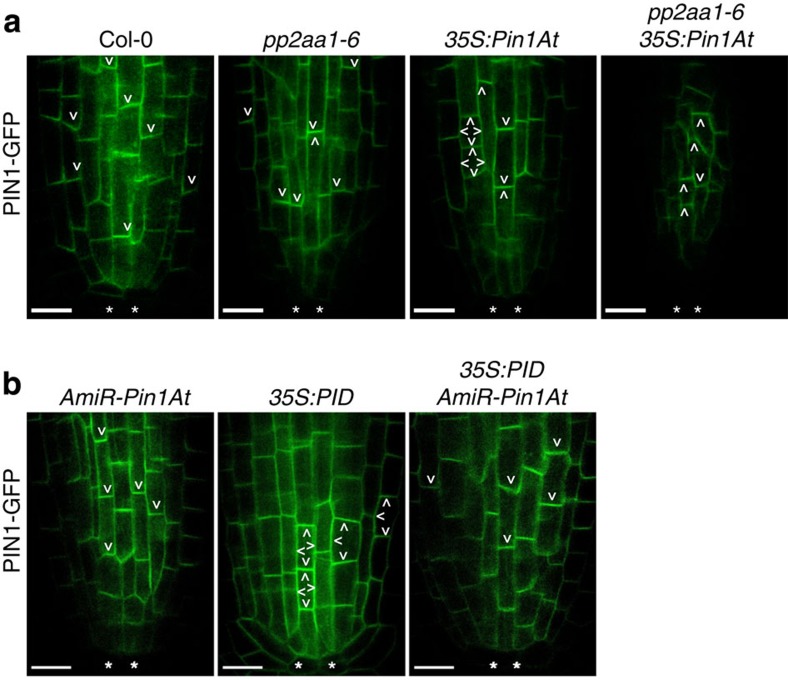
***Pin1At*** **affects polar localization of PIN1.** (**a**,**b**) Visualization of PIN1-GFP subcellular localization in primary roots of wild-type, *pp2aa1-6*, *35S:Pin1At*, *pp2aa1-6 35S:Pin1At* (**a**), *AmiR-Pin1At*, *35S:PID* and *35S:PID AmiR-Pin1At* (**b**) seedlings at 3 DAG by confocal laser scanning microscopy (CLSM). PIN1-GFP polarity is indicated by arrows. **Quiescent centre cells. Scale bars, 10 μm.

As PID kinase and PP2A phosphatases antagonistically regulate PIN phosphorylation to control auxin transport^14^,^15^,^32^,^33^,^38^, we next examined whether *Pin1At* are involved in modulating PIN proteins. PIN proteins function as auxin efflux facilitators and their asymmetric apical-basal targeting at the PM determines the rate and direction of auxin flux^13^. PIN1, 2, 3, 4 and 7 have been reported to directly or indirectly regulate root gravitropism^39^,^40^,^41^,^42^,^43^,^44^,^45^,^46^, whereas PIN1 and PIN2 play predominant roles in mediating rootward and shootward auxin transport, respectively. Considering that rootward auxin transport was severely affected in *pp2aa1-6 35S:Pin1At* roots, we first examined the effect of *Pin1At* on the subcellular localization of PIN1-GFP in *pPIN1:PIN1-GFP*^47^ with different genetic backgrounds (Fig. 5a). In wild-type root apical tissues, PIN1-GFP was detected mainly in the basal membrane (rootward side) of stele cells. In *pp2aa1-6* roots, basal PIN1-GFP localization was still observed, although the intensity of PIN1-GFP signals was reduced in stele cells. In contrast, the basal subcellular polarity of PIN1-GFP became less pronounced in most stele cells of the *35S:Pin1At* roots examined and the PIN1-GFP protein abundance also decreased in these cells (Fig. 5a). Reduction of the subcellular polarity of PIN1-GFP was similarly observed in *pp2aa1-6 35S:Pin1At* roots, which showed a further decrease in the PIN1-GFP protein abundance (Fig. 5a). Quantification of PIN1-GFP intensity in stele cells revealed that the ratio of PIN1-GFP intensity at polar (apical-basal) versus lateral PM significantly decreased in *35S:Pin1At* and *pp2aa1-6 35S:Pin1At* (Supplementary Fig. 10), further demonstrating the disruption of PIN1-GFP polarity in the *35S:Pin1At* background.

As *AmiR-Pin1At* restored *DR5:GUS* expression in *35S:PID* root tips (Supplementary Fig. 7), we further checked PIN1-GFP localization in *AmiR-Pin1At*, *35S:PID* and *35S:PID AmiR-Pin1At* roots. In agreement with a previous report^33^, constitutive expression of *PID* caused a basal-to-apical shift of PIN1 polarity in stele cells (Fig. 5b). However, downregulation of *Pin1At* in *35S:PID* led to basal PIN1-GFP localization in ∼70% of *35S:PID AmiR-Pin1At* roots (Fig. 5b). These results together with the observation of partial co-localization of Pin1At-GFP and PIN1*-*mCherry in root stele cells (Supplementary Fig. 11) suggest that *Pin1At* affects the subcellular polarity of PIN1 protein and negatively regulates auxin transport.

In contrast to the effect of *Pin1At* on PIN1, we did not observe significant changes in PIN2-GFP subcellular localization in epidermis and cortex cells in *AmiR-Pin1At* and various genotypes bearing *35S:Pin1At* (Supplementary Fig. 12). We also examined whether *Pin1At* affects the expression patterns of PIN3 and PIN7 in roots, as both of them are expressed in gravity-sensing root columella cells and play redundant roles in the regulation of auxin distribution and root gravitropism^42^,^43^,^48^. The overall localization patterns of PIN3-GFP and PIN7-GFP in stele and columella cells were mostly similar in *35S:Pin1At* and wild-type roots (Supplementary Fig. 13).

### Pin1At catalyses isomerization of Ser/Thr-Pro motifs in PIN1

**Figure 6:**
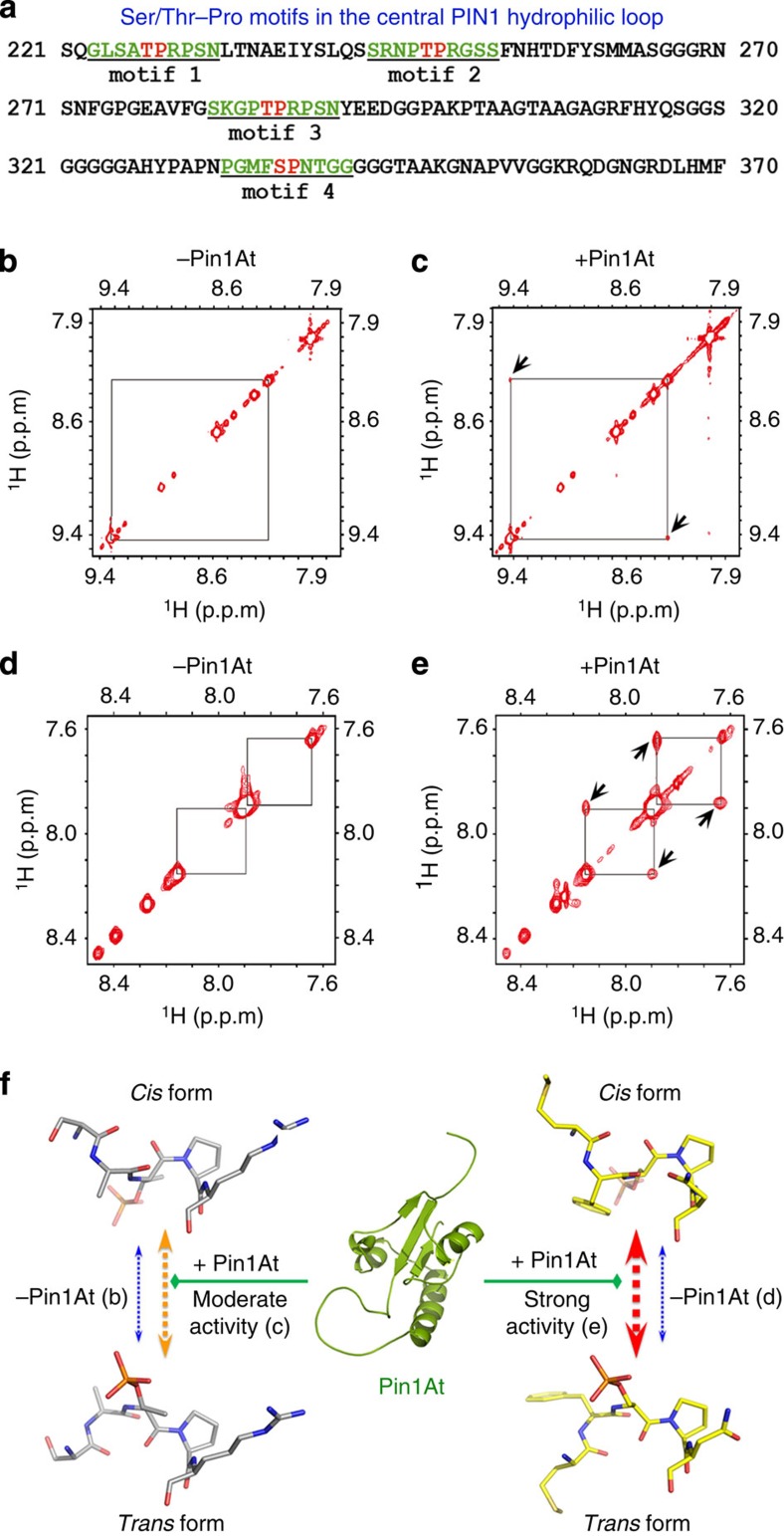
Pin1At catalyses *cis/trans* isomerization of pSer/Thr-Pro motifs in PIN1. (**a**) The amino acid sequences of the central PIN1 hydrophilic loop contain four SP or TP motifs that are potentially catalysed by Pin1At. (**b**–**e**) Selected regions of two-dimensional ROESY spectra of the phosphorylated PIN1 peptides in the absence (**b**,**d**) or presence (**c**,**e**) of Pin1At at a mixing time of 110 ms. Exchange cross-peaks resulting from Pin1At-catalysed *cis/trans* isomerization of pSer/Thr-Pro motifs are indicated by arrows. The NMR results for the peptides corresponding to motif 1 (**b**,**c**) and motif 4 (**d**,**e**) are shown. (**f**) Structural model represents the different catalytic effects of Pin1At on the phosphorylated PIN1 motifs examined. In the absence of Pin1At (in blue), the conformational changes between *cis* and *trans* are very slow. However, Pin1At moderately catalyses the conformational changes of three pThr-Pro bonds (motifs 1-3; in orange) and strongly catalyses one pSer-Pro bond (motif 4; in red).

Previous studies have revealed that several conserved motifs in the PIN1 protein mediate PIN1 polarity and auxin transport through reversible phosphorylation^16^,^17^. These motifs include three TPRXS(N/S) and one SPNT in the central PIN1 hydrophilic loop (Fig. 6a). Functional studies on loss-of-phosphorylation and phosphomimic PIN1-GFP variants have suggested that reversible phosphorylation of these motifs is necessary and sufficient to change PIN1 polarity and auxin flow^16^,^17^. As Pin1At specifically catalyses the *cis-trans* isomerization of the phosphorylated Ser/Thr residues preceding proline (pSer/Thr-Pro) in *Arabidopsis*^19^,^22^, we examined whether Pin1At catalysed the *cis/trans* conformational change of the phosphorylated Ser/Thr-Pro motifs in the above-mentioned PIN1 hydrophilic loop (Fig. 6a) at per residue resolution by nuclear magnetic resonance (NMR) spectroscopy.

We synthesized the phosphorylated peptides corresponding to the Ser/Thr-Pro motifs 1-4 in the central PIN1 hydrophilic loop (Fig. 6a) and determined the effect of Pin1At on the conformational exchanges of these peptides using homonuclear two-dimensional 1H-1H rotating-frame Overhauser effect spectroscopy NMR (ROESY) as reported previously^22^,^49^. In the absence of Pin1At, we did not observe exchange peaks in the ROESY spectra of the phosphorylated PIN1 peptides (Fig. 6b,d), indicating that the exchange between the *cis* and *trans* conformations is too slow to be detected on the NMR timescale (<0.1 s^−1^). In contrast, in the presence of Pin1At, we detected moderate exchange peaks for the peptides derived from motif 1 (Fig. 6c arrows), and motifs 2 and 3 (Supplementary Fig. 14), and the strongest exchange peaks for the peptide of the motif 4 (Fig. 6e arrowheads). As expected, we did not detect exchange peaks for all four non-phosphorylated peptides derived from motifs 1-4 (Supplementary Fig.15). These findings demonstrate that Pin1At catalyses the *cis/trans* conformational change of the four phosphorylated Ser/Thr-Pro peptides in the PIN1 hydrophilic loop (Fig. 6f), implying that these four Ser/Thr-Pro motifs are Pin1At substrates *in vitro*.

Consistent with the strongest exchange peaks found for the peptide of the motif 4 harbouring SPNT motif (Fig. 6a), manipulation of PIN1 phosphorylation at Ser337/Thr340 within this motif has been reported to alter PIN1 polarity and auxin transport^17^. We thereafter examined Pin1At effect on PIN1-GFP subcellular localization in root stele cells using two mutant versions, PIN1-GFP (Ala) and PIN1-GFP (Asp), in which Ser337 and Thr340 were both converted into Ala (a non-phosphorylation mimic) and to Asp (a phosphorylation mimic), respectively^17^. PIN1-GFP (Ala) remained a predominant basal localization in *35S:Pin1At* (Supplementary Fig. 16) as compared with a reduced basal subcellular polarity of PIN1-GFP in *35S:Pin1At* (Fig. 5a). Furthermore, phosphomimic PIN1-GFP (Asp) displayed obvious non-polar localization or enhanced basal-to-apical shift in wild-type stele cells. This abnormal localization was almost completely suppressed in *AmiR-Pin1At* (Supplementary Fig. 16). These observations suggest that Pin1At effect on PIN1 subcellular localization is mediated by PIN1 phosphorylation at Ser337/Thr340 located within the main Ser/Thr-Pro motif through which Pin1At specifically catalyses the *cis-trans* isomerization of PIN1.

PIN1-type PPIase plant homologues, including Pin1At, contain a PPIase domain with four additional amino acids, which distinguish them from PIN1-type homologues in other living organisms^22^,^50^. As the four additional amino acids have been shown to mediate the proper function of Pin1At in the control of flowering time^22^, we also examined whether they are important for Pin1At role in controlling root gravitropism. We overexpressed the mutated *Pin1At* (*35S:mPin1At*), in which the four extra amino acids were deleted, in wild-type or *pp2aa1-6* background. We identified five lines each for *35S:mPin1At* or *pp2aa1-6 35S:mPin1At* plants, all of which contained single insertion of the *35S:mPin1At* transgene. Examination of their root phenotypes did not reveal obvious changes in root gravitropic responses compared with those of either wild-type or *pp2aa1-6* plants, respectively (Fig. 2a and Supplementary Fig. 17). This is in contrast to the effect of *35S:Pin1At* on root gravitropism (Figs 1a and 2b), indicating that the additional four amino acids in the PPIase domain are critical for Pin1At to exert its role in modulating root gravitropic responses.

## Discussion

**Figure 7:**
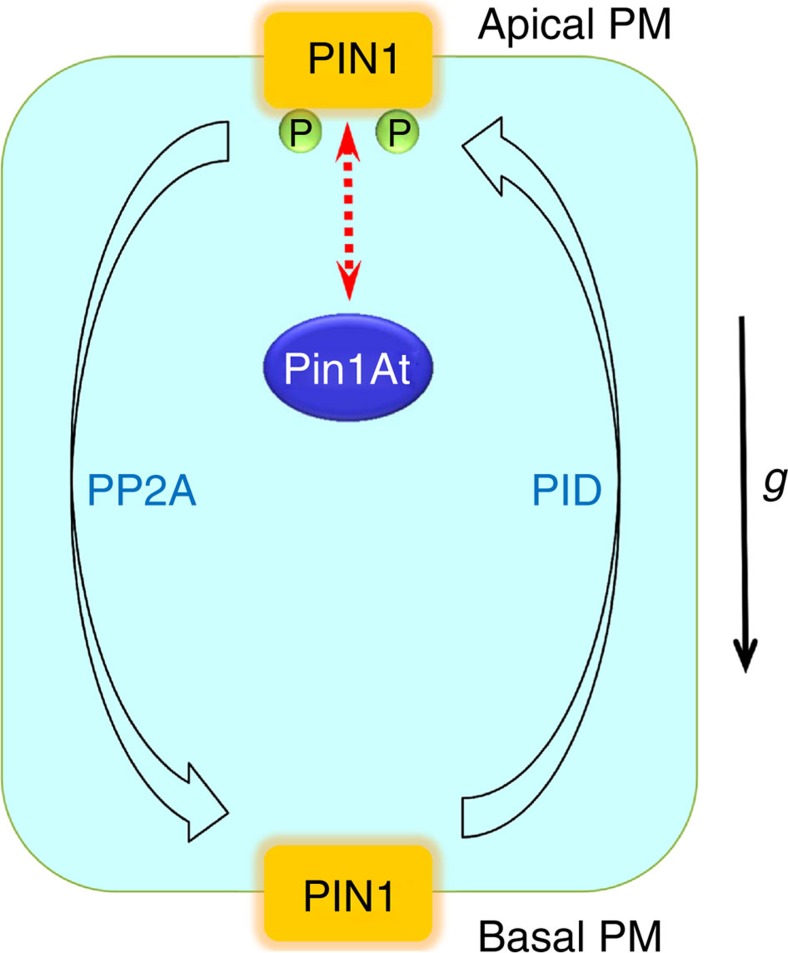
A proposed model of regulation of PIN1 subcellular localization and root gravitropism by Pin1At. Reversible phosphorylation of PIN1 by PID and PP2A mediates apical (shootward) or basal (rootward) polar localization of PIN1 and contributes to root gravitropic responses. Pin1At accelerates the *cis/trans* conformational change of the phosphorylated Ser/Thr-Pro motifs in the central PIN1 hydrophilic loop and affects polar localization of PIN1 in the PM of root stele cells mediated by PID and PP2A. Consistently, knockdown or overexpression of *Pin1At* largely suppresses or enhances the root agravitropic responses of *pp2aa1 pp2aa2* and *35S:PID* seedlings, respectively. Thus, *cis/trans* isomerization of PIN1 by Pin1At affects the antagonistic effects of PP2A and PID on the polar localization of PIN1, which correlates with regulation of root gravitropism.

Since the discovery of the involvement of directional transport of auxin in gravitropic response by Cholodny and Went more than 80 years ago^51^, many studies have been carried out and confirmed the importance of auxin in mediating the tropic growth responses. Auxin transport efflux carriers such as PINs have emerged as key regulators that determine the directional auxin flow and root gravitropic growth through their asymmetric (polar) subcellular distribution^13^. Reversible phosphorylation of PINs by PID kinase (and possibly other AGC kinases) and PP2A phosphatases antagonistically regulates PIN polar localization, thus controlling intracellular auxin flux and root gravitropism^14^,^16^,^17^. In this study, we report that Pin1At, an *Arabidopsis* PPIase, catalyses the *cis/trans* isomerization of PIN1 and modulates its polar subcellular localization in root stele cells mediated by PID and PP2As, which correlates to regulation of root gravitropism (Fig. 7).

Among three PP2A subunit isoforms in *Arabidopsis*, PP2AA1 plays a dominant role in regulating root gravitropism^14^,^15^. The agravitropic phenotype of *pp2aa1-6* is enhanced by *35S:Pin1At* (Fig. 2a,b), while *AmiR-Pin1At* suppresses the agravitropic defect of *pp2aa1-6* and *pp2aa1-6 pp2aa2-1* (Fig. 2c–e). Meanwhile, knocking down *Pin1At* fully rescues the collapsed root meristems and considerably suppresses the root agravitropic phenotype caused by *35S:PID* (Fig. 3b). These observations suggest that Pin1At genetically functions in balancing the opposite effects of PP2A and PID on the regulation of root gravitropism.

PID and PP2A act antagonistically to regulate reversible phosphorylation of PIN1 and its apical or basal polar localization. Phosphorylation of PIN1 increases in both *35S:PID* and *pp2aa1-6*, which causes a basal-to-apical polarity shift in PIN subcellular localization and consequently contributes to the root agravitropic phenotype^14^,^15^,^32^,^33^,^38^. Consistent with the genetic effect of Pin1At on the root agravitropic phenotype of *35S:PID* and *pp2aa1-6*, Pin1At affects both PIN1 polarity in root stele cells (Fig. 5) and auxin transport (Fig. 4b and Supplementary Fig. 9), both of which are regulated by PID and PP2A. As Pin1At accelerates the *cis/trans* conformational change of the phosphorylated Ser/Thr-Pro motifs in the central PIN1 hydrophilic loop (Fig. 6), the conformational dynamics of PIN1 regulated by Pin1At, which allows relaxation of local energetically unfavourable Ser/Thr-Pro conformational states, could play an important role in mediating the antagonistic effects of PID and PP2A on polar localization of PIN1. Interestingly, the Pin1At orthologue in human, Pin1, has been shown to mediate the phosphorylation signalling partially through regulating dephosphorylation of several proteins involved in mitosis by PP2A^52^. Thus, Pin1At-like PPIases might be evolutionarily conserved enzymes that catalyse conformational changes of key substrates relevant to reversible protein phosphorylation involving PP2As and kinases in both plants and animals.

Among several Ser/Thr-Pro motifs in the central PIN1 hydrophilic loop that have been shown to mediate PIN1 polarity and auxin transport^16^,^17^, NMR analyses have detected strong exchange cross-peaks for the peptide 4 that contains the (p)SPNT motif, but weak peaks for other three peptides harbouring (p)TPRXS motifs in the presence of Pin1At (Fig. 6), suggesting that Pin1At could mainly target the (p)SPNT motif for catalysing the *cis/trans* conformational change of PIN1. It is noteworthy that the SPNT motif is not present in other PM localized PIN1-type proteins, such as PIN2, 3, 4 and 7 (ref. 16). This may partly explain why the effect of Pin1At appeared to be specific to PIN1, rather than other PIN1-type efflux carriers that directly or indirectly regulate root gravitropism. Consistently, among PINs examined, only polar localization of PIN1 is changed in *35S:Pin1At* root cells, which is correlated with compromised rootward auxin transport and the reduced auxin maximum at the root tip.

As the SPNT motif might not be a direct target of PID^16^,^17^, the strongest effect of Pin1At on isomerization of (p)SPNT implies that other kinases could play a more direct role in the regulation of phosphorylation events relevant to conformational changes in the central PIN1 hydrophilic loop. It has been suggested that PID and its homologues WAG1 and WAG2, in the same AGCVIII kinase subfamily, play redundant roles in phosphorylating PIN proteins at TPRXS(N/S) motifs to direct PIN polarity and auxin transport^53^. In addition, recent studies have demonstrated that another subfamily of AGCVIII kinases comprising four functionally redundant members, namely D6 PROTEIN KINASE (D6PK), D6PK-LIKE1 (D6PKL1), D6PKL2 and D6PKL3, is also involved in the phosphorylation of PIN1 (refs. 54, 55). However, whether the SPNT motif is the phosphosite targeted by WAG1, WAG2 or D6PKs is so far unknown. As PIN1 phosphorylation could be controlled by multiple kinases targeting to different phosphosites of PIN1 in different developmental context, further identification of specific kinase(s) for the SPNT motif would shed light on the regulatory network in which plants coordinate the regulation of conformational dynamics of PIN1 by Pin1At and other regulatory events involved in PIN1-mediated auxin efflux.

In addition to PIN1, our results imply that Pin1At may also affect other unknown targets involved in the regulation of root gravitropism. First, the change in shootward auxin transport (Supplementary Fig. 9) could also contribute to the root agravitropic phenotype of *35S:Pin1At*. Although PIN2 plays an important role in regulating shootward auxin transport and root gravitropism^44^,^45^,^46^,^56^, subcellular localization of PIN2-GFP in epidermis and cortex cells in *AmiR-Pin1At* and *35S:Pin1At* is not significantly changed (Supplementary Fig. 12), indicating that alteration in shootward auxin transport in *35S:Pin1At* could depend on other auxin transporters. Second, as columella cells contain amylopasts that sense the gravity stimulus, a decreased population of gravity-sensing columella cells in *35S:Pin1At* (Fig. 1b and Supplementary Fig. 2) could also contribute to the reduced gravity response exhibited by *35S:Pin1At*. It would be interesting to further identify other yet undiscovered target(s) of Pin1At that may mediate the generation of columella cells, thus affecting root gravitropism.

## Methods

### Plant material and growth conditions

All mutants and transgenic lines employed in this study are in the *Arabidopsis thaliana* ecotype Columbia (Col-0) background. Those previously described include *35S:Pin1At*^22^, *pp2aa1-6* or *rcn1-6* (SALK_059903)^31^, *pp2aa2-1* (SALK_042724)^15^, *pp2aa3-1* (SALK_014113)^15^, *35S:PID*^32^, *pPIN1:PIN1-GFP*^47^, *pPIN2:PIN2-GFP*^57^, *pPIN3:PIN3-GFP*^58^, *pPIN7:PIN7-GFP*^48^, *pPIN1:PIN1-GFP (Ala)*^17^, *pPIN1:PIN1-GFP (Asp)*^17^ and *35S:DII-VENUS*^37^. *AmiR-Pin1At*, *Pin1At-GUS*, *Pin1At-GFP* and *35S:mPin1At* were generated in this study through *Agrobacterium tumefaciens*-mediated transformation. Surface-sterilized seeds were sown on 1 × MS (Sigma M5524) agar medium containing 1% (w/v) sucrose and 0.8% (w/v) Agar (Sigma A1296). The plates were kept at 4 °C in darkness for 3 days (stratification) and then transferred to a tissue culture room set at 22 °C under long days (16 h light/8 h dark). Plants were thereafter grown on vertically oriented plates and pictures were taken 4 days after germination (4 DAG) unless otherwise indicated. NPA treatment was performed by growing seedlings on MS medium supplemented with 0.3 μM NPA (Sigma).

### Plasmid construction

To construct *Pin1At-GUS-P1*, a 550-bp *Pin1At* 5′-upstream sequence was amplified using primers Pin1At-F and Pin1At-P1-R, and cloned into pHY107 (ref. 59). To construct *Pin1At-GUS-P2*, a 1.7-kb *Pin1At* genomic fragment including the 550-bp upstream sequence and the full coding sequence plus the intron was amplified using the primers Pin1At-F and Pin1At-P2-R, and cloned into pHY107. To construct *Pin1At-GUS-P3* and *pPin1At:Pin1At-GFP*, two cloning steps were adopted. First, a 2.6-kb *Pin1At* genomic fragment including the 550-bp upstream sequence, the 1.2-kb coding sequence plus the intron and the 900-bp 3′-untranslated region was amplified using the primers Pin1At-F and Pin1At-P3-R, and cloned into pHY105 (ref. 59), to generate pHY105-gPin1At. Second, the *GUS* or *GFP* sequence was amplified using the primer pairs Pin1At-GUS-F/Pin1At-GUS-R or Pin1At-GFP-F/Pin1At-GFP-R, respectively, and inserted just before the stop codon of *Pin1At* in the pHY105-gPin1At construct using a modified QuikChange Site-Directed Mutagenesis approach^60^. These PCR fragments were annealed to the methylated template plasmid DNA containing pHY105-gPin1At and elongated with the Phusion DNA polymerase (Thermo Scientific). On *Dpn*I digestion, the mutated plasmids containing either *GUS* or *GFP* were recovered from *Escherichia coli* transformation. *pPIN1:PIN1-mCherry* was generated by replacing *GFP* with *mCherry* in *pPIN1:PIN1-GFP*^61^.

To construct *AmiR-Pin1At*, design of the artificial microRNA was performed using the software on the website (http://wmd2.weigelworld.org). Based on the *Pin1At* sequence, a set of four primers (AmiR-Pin1At-I/II/III/IV) were generated and used for the PCR amplification according to the published protocol^26^. The resulting PCR fragment was cloned into a pGreen-35S vector^62^. All primers for plasmid construction are listed in Supplementary Table 1.

### Root gravitropism assay

Vertically grown seedlings at 4 DAG were transferred to the assay MS plates and reoriented at 90°. Pictures were taken 24 h after gravi-stimulation. For the time-course root gravitropism assay, surface-sterilized seeds were plated onto MS agar medium and then overlaid with the same medium. Seedlings at 4 DAG were reoriented by 90° and root tip angles were measured 2, 4, 6, 12 and 24 h after reorientation.

### Expression analysis

Total RNA from root tissues was isolated using the RNeasy Plant Mini Kit (Qiagen) and reverse transcribed with iScript Reverse Transcription Supermix for RT-qPCR System (Bio-Rad) according to the manufacturers' instructions. Real-time PCR was performed in triplicates on CFX384 Touch Real-Time PCR Detection System (Bio-Rad) with iQ SYBR Green Supermix (Bio-Rad). The difference between the cycle threshold (Ct) of the target gene and the Ct of *TUB2* (ΔCt=Ct_target gene_−Ct_tubulin_) was used to obtain the normalized expression of a target gene, which corresponded to 2^−ΔCt^. Expression analysis was performed on at least three biological replicates. Primers for real-time PCR are listed in Supplementary Table 2.

### Reporter-based auxin transport assay

Auxin transport assay using the *DR5:GUS* reporter was performed according to a published protocol^34^. Briefly, vertically grown seedlings at 5 DAG were transferred to the assay MS plates with the root-shoot junctions aligned for rootward auxin transport assay or with root apices aligned for shootward auxin transport assay. IAA, NPA or MOCK solution was prepared in agar droplets. For rootward assay, droplets were positioned directly below root-shoot junctions and the transport was allowed to proceed for 9 h in the dark condition at room temperature. Thereafter, the seedlings were subjected to histochemical GUS staining for 15 h at 37 °C. For shootward assay, droplets were placed just below root apices and covered the root tips by around 0.5 mm, and the transport was allowed to proceed for 5 h in the dark condition at room temperature. Incubation time for GUS staining was just 2 h to avoid overstaining. To examine rootward auxin distribution by the DII-VENUS reporter^37^, IAA, NPA or MOCK agar droplets were applied to root-shoot junctions for 3 h.

### Confocal microscopy

Fluorescent signals including GFP, mCherry and VENUS in roots were visualized in water in the presence or absence of propidium iodide without fixation and imaged using a confocal laser scanning microscopy (Leica TCS SP5X). Leica Application Suite Advanced Fluorescence software was used to process images and to quantify the fluorescent intensity of PIN1-GFP at the PM.

### NMR analysis

All NMR experiments were performed on a Bruker 800 MHz NMR spectrometer at 25 °C (refs 22, 49, 63). Briefly, all spectra were recorded on peptide samples containing 20 mM phosphate buffer (90% H_2_O and 10% D_2_O pH 6.5) in the presence or absence of Pin1At. The peptide concentration was 2.4 mM and the Pin1At concentration was 0.03 mM for the samples of phosphorylated and non-phosphorylated PIN1 peptides (GLSA[p]TPRPSN, SRNP[p]TPRGSS, SKGP[p]TPRPSN and PGMF[p]SPNTGG; Fig. 6a).

## Additional information

**How to cite this article:** Xi, W. *et al.* Pin1At regulates PIN1 polar localization and root gravitropism. *Nat. Commun.* 7:10430 doi: 10.1038/ncomms10430 (2016).

## Supplementary Material

Supplementary Information Supplementary Figures 1-17 and Supplementary Tables 1-2
