# Supplementary material for: Pin1At regulates PIN1 polar localization and root gravitropism
Source: Nat Commun. 2016 Jan 21;7:10430. doi: 10.1038/ncomms10430 (PMC4736118; doi:10.1038/ncomms10430)
Supplement: Supplementary Information — Supplementary Figures 1-17 and Supplementary Tables 1-2 [file ncomms10430-s1.pdf]

# SUPPLEMENTARY INFORMATION

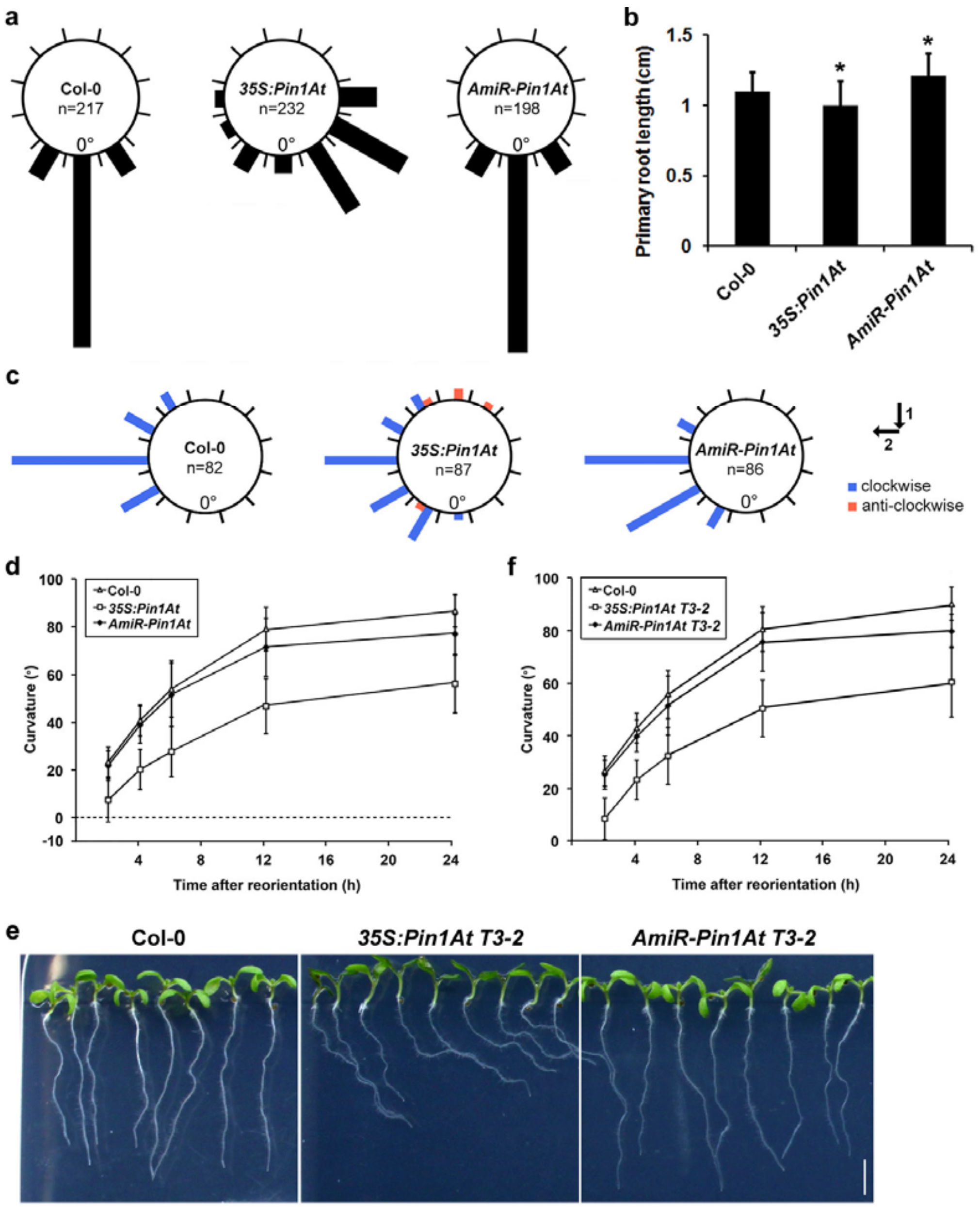


**Supplementary Figure 1. Root phenotypes of wild-type Col-0, *35S:Pin1At* and *AmiR-Pin1At* plants.** (**a**) Quantitative analysis of growth directions of the primary roots in wild-type, *35S:Pin1At* and *AmiR-Pin1At* plants shown in Fig. 1a. The direction of root growth was measured by assigning to one of the twelve 30° sectors on a gravitropism diagram. The length of each bar in the diagram represents the percentage of seedlings showing the direction of root growth within the respective sector. n is the total number of seedlings analyzed for each genotype. (**b**) Comparison of primary root length of wild-type, *35S:Pin1At* and *AmiR-Pin1At* plants at 4 DAG. Values are mean ± SD of at least 35 independent roots. Asterisks (*) indicate statistically significant differences in primary

root length in transgenic plants compared with wild-type plants (two-tailed paired Student's *t* test, $P < 0.05$). (**c**) Quantitative analysis of root gravitropic re-orientation responses of wild-type, *35S:Pin1At* and *AmiR-Pin1At* plants shown in Fig. 1c. Vertically grown seedlings at 4 DAG were turn 90°. Root tip positions before and 24h after gravistimulation were determined. A blue or red bar represents the percentage of seedlings growing in a clockwise or anti-clockwise direction to the new gravity vector (arrow 2), respectively. n is the total number of seedlings analyzed for each genotype. Arrows indicate the vector of gravity before (1) and after (2) gravistimulation. (**d**) Kinetics of root reorientation of wild-type, *35S:Pin1At* and *AmiR-Pin1At* seedlings in response to gravistimulation. Vertically grown seedlings at 4 DAG embedded in 0.8% agar plates were reoriented by 90° and root tip angles were recorded 2, 4, 6, 12 and 24 h after gravistimulation. Values are mean ± SD of at least 100 independent roots. There are statistically significant differences in root curvature angles at all time points examined between *35S:Pin1At* and wild-type plants, and at 12 h and 24 h between *AmiR-Pin1At* and wild-type plants (two-tailed paired Student's *t* test, $P < 0.01$). (**e**) Root phenotype of wild-type Col-0 and other two independent transgenic lines (*35S:Pin1At T3-2* and *AmiR-Pin1At T3-2* plants) at 4 DAG. Scale bar, 2.5 mm. (**f**) Kinetics of root reorientation of wild-type, *35S:Pin1At T3-2* and *AmiR-Pin1At T3-2* seedlings in response to gravistimulation. There are statistically significant differences in root curvature angles at all time points examined between *35S:Pin1At T3-2* and wild-type plants, and at 12 h and 24 h between *AmiR-Pin1At T3-2* and wild-type plants (two-tailed paired Student's *t* test, $P < 0.01$).

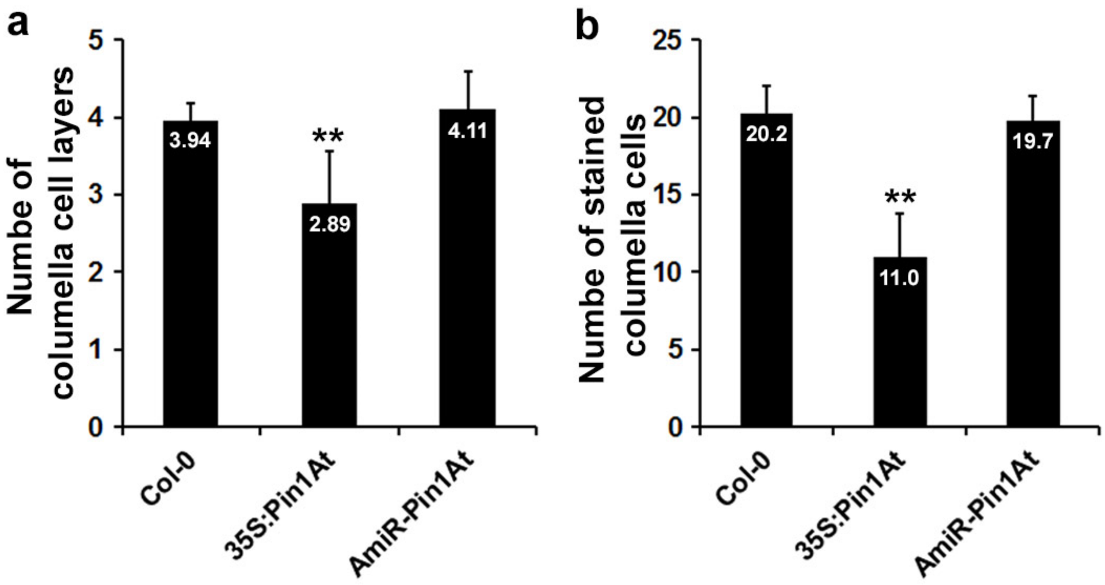


**Supplementary Figure 2. Quantification of columella cells after Lugol's staining of root tips of wild-type, *35S:Pin1At* and *AmiR-Pin1At* seedlings at 4 DAG.** (**a**) Number of columella cell layers in various genotypes. (**b**) Number of stained columella cells in various genotypes. Values are mean ± SD of at least 20 independent roots. Asterisks (**) indicate statistically significant differences in transgenic plants compared with wild-type plants (two-tailed paired Student's *t* test, $P < 0.01$).

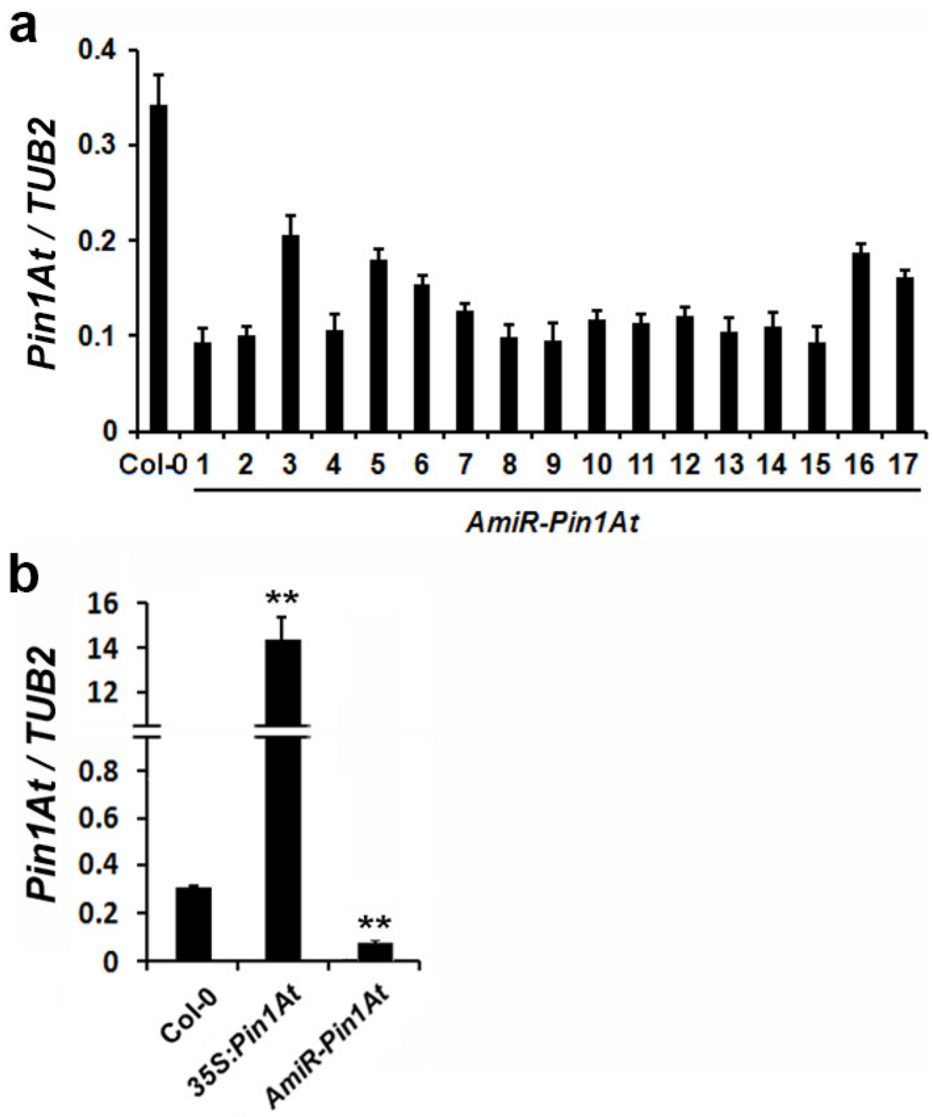


**Supplementary Figure 3. Examination of *Pin1At* expression in various transgenic plants. (a)** Downregulation of *Pin1At* in *AmiR-Pin1At* transgenic lines. The artificial microRNA targeting the 3' region of the *Pin1At* transcript successfully knocks down *Pin1At* mRNA levels in 17 independent *AmiR-Pin1At* transgenic lines at the T3 generation. There are statistically significant differences in gene expression levels between all *AmiR-Pin1At* transgenic lines examined and wild-type plants (two-tailed paired Student's *t* test, $P < 0.01$). **(b)** Quantitative expression of *Pin1At* in the roots of wild-type, *35S:Pin1At* and *AmiR-Pin1At* plants at 4 DAG. Gene expression was normalized against the expression of *TUB2*. Error bars denote SD. Asterisks (**) indicate significant differences in gene expression levels in transgenic plants compared to those in wild-type plants (two-tailed paired Student's *t* test, $P < 0.01$).

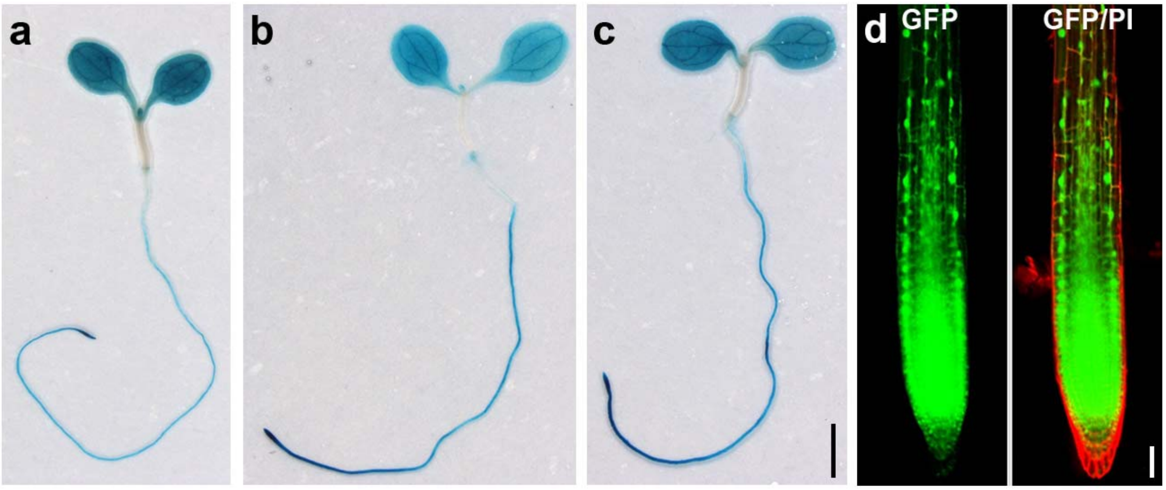


**Supplementary Figure 4. Expression patterns of *Pin1At* in seedlings. (a-c)** GUS staining of *Pin1At-GUS-P1* (**a**), *Pin1At-GUS-P2* (**b**), and *Pin1At-GUS-P3* (**c**) seedlings shows *Pin1At* expression predominantly in the root, root-shoot junction, and cotyledons. Scale bar, 5 mm. (**d**) Localization of Pin1At-GFP in the primary root of *gPin1At-GFP*. PI, propidium iodide. Scale bar, 50 μm.

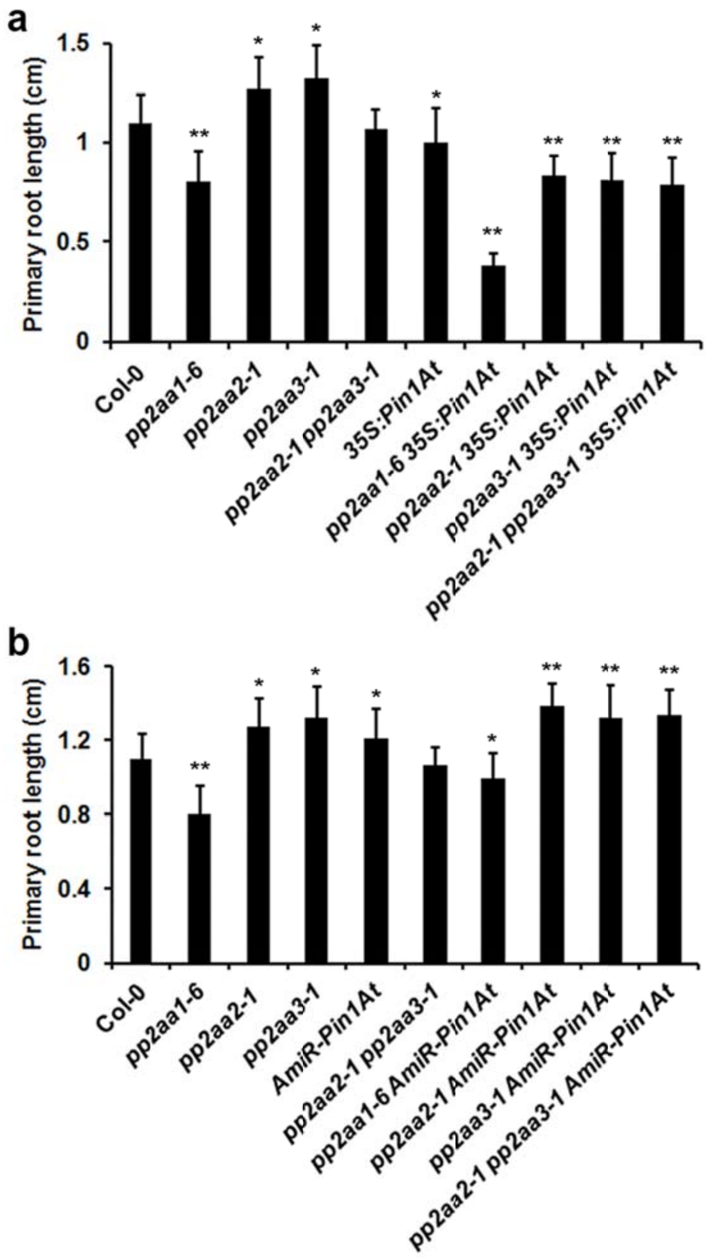


**Supplementary Figure 5. Primary root length of the seedlings in *pp2aa* mutant backgrounds.** (**a,b**) Comparison of primary root length of *35S:Pin1At* (**a**) or *AmiR-Pin1At* (**b**) related seedlings at 4DAG. Each value represents the average ± SD for at least 40 seedlings. Asterisks indicate significant differences in root length in transgenic plants compared to that in wild-type plants (two-tailed paired Student's *t* test, *$P < 0.05$, **$P < 0.01$).

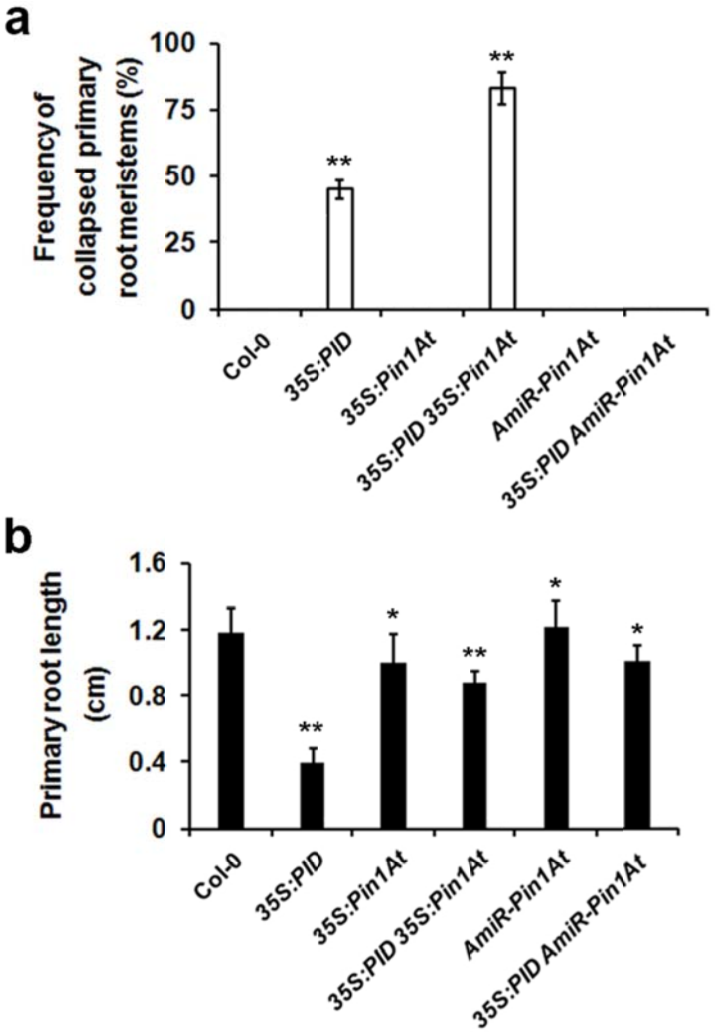


**Supplementary Figure 6. Quantitative analyses of root development in *35S:PID*-related seedlings.** (**a,b**) Comparison of frequency of collapsed primary root meristems (**a**) and primary root length (**b**) of *35S:PID*-related seedlings at 4DAG. Each value represents the average ± SD for at least 40 seedlings. Asterisks indicate significant differences in transgenic plants compared to wild-type plants (two-tailed paired Student's $t$ test, $*P < 0.05$, $**P < 0.01$).

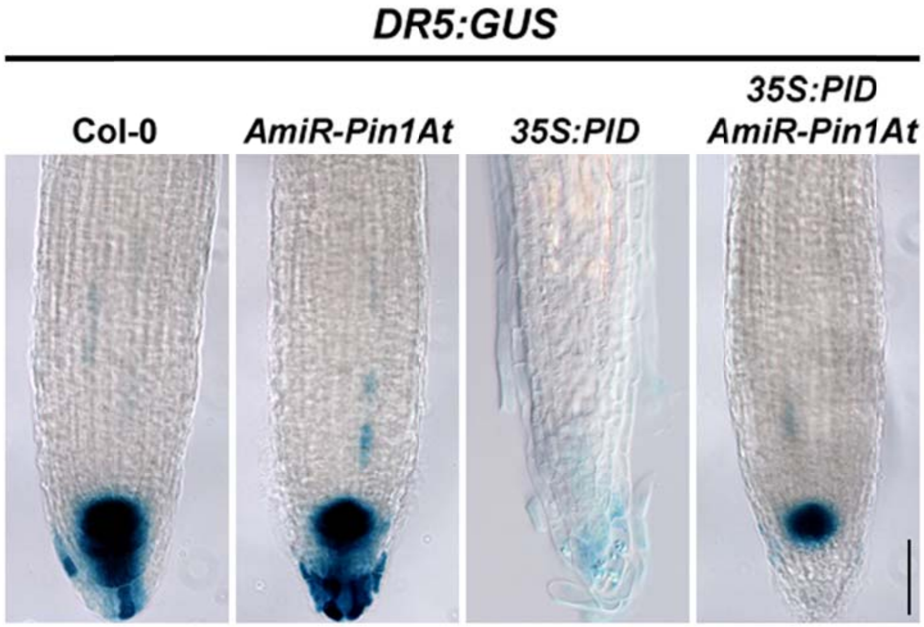


**Supplementary Figure 7. Restoration of *DR5:GUS* expression in *35S:PID* root tips by *AmiR-Pin1At* for the rootward auxin transport assay.** Scale bar, 50 µm.

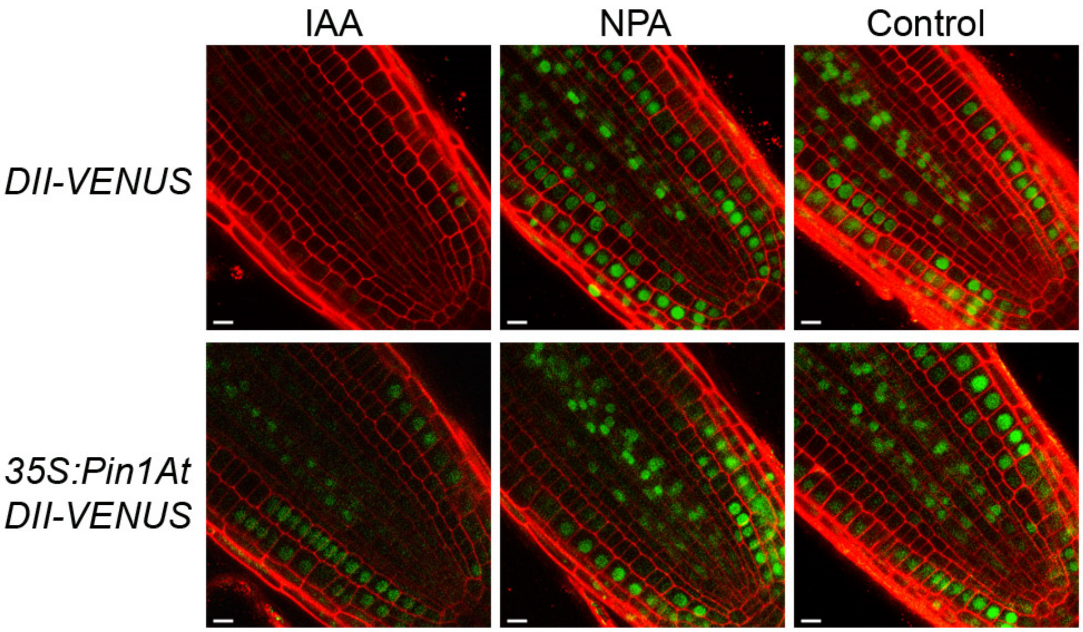


**Supplementary Figure 8. Effect of *35S:Pin1At* on DII-VENUS fluorescence in root tips in response to various treatments at root-shoot junctions.** Root tips treated by 1 µM IAA and 1 µM NPA, or MOCK-treated for 3 h were visualized by CLSM. Scale bars, 10 µm.

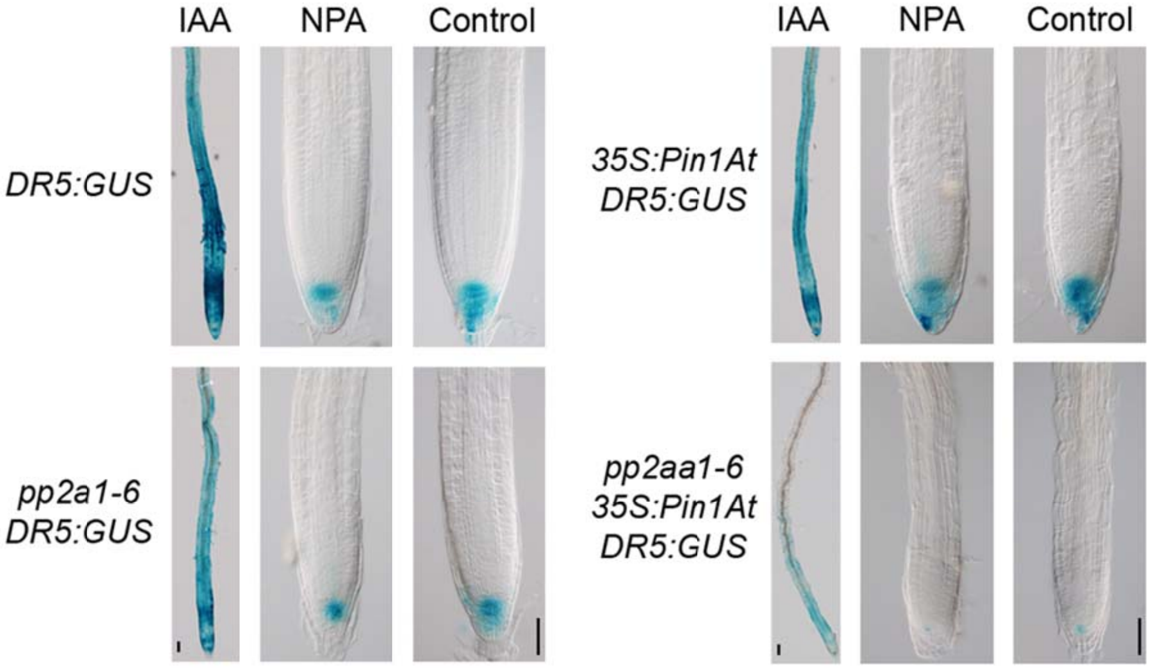


**Supplementary Figure 9. Comparison of the auxin-inducible *DR5:GUS* reporter gene expression in root tips for the shootward auxin transport assay.** IAA, NPA, or MOCK-treated root tips were subjected to GUS staining to visualize shootward auxin transport. Scale bars, 50 µm.

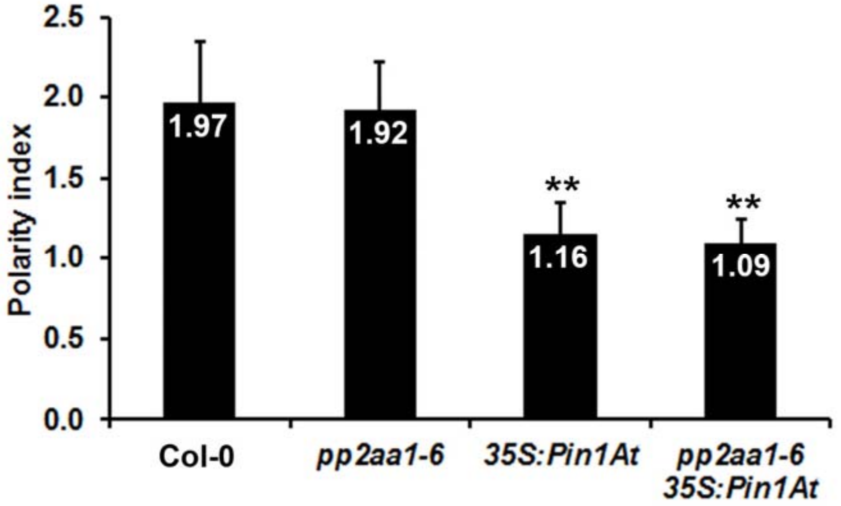


**Supplementary Figure 10. Quantification of PIN1-GFP polarity in stele cells of wild-type, *pp2aa1-6, 35S:Pin1At*, and *pp2aa1-6 35S:Pin1At* at 3 DAG.** The fluorescent intensity of PIN1-GFP at the polar and lateral plasma membrane of cells was measured by Leica Application Suite Advanced Fluorescence (LAS AF) software. Polarity index was determined by the ratio of PIN1-GFP intensity at polar versus lateral plasma membrane. The value represents the average ± SD for 40 stele cells from 5 roots for each genotype. Asterisks (**) indicate statistically significant differences in polarity index in *35S:Pin1At* and *pp2aa1-6 35S:Pin1At* compared with that in wild-type plants (two-tailed paired Student’s *t* test, $P < 0.01$).

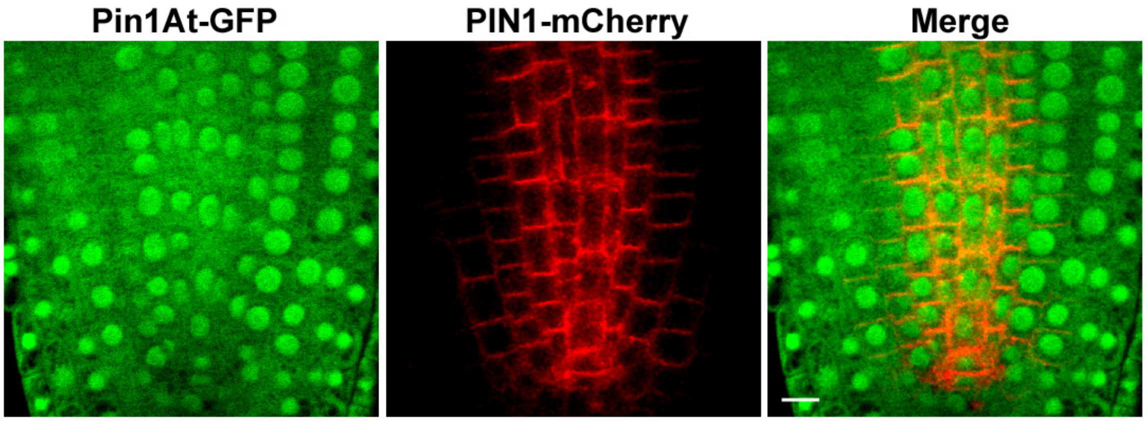


**Supplementary Figure 11. Visualization of Pin1At-GFP and PIN1-mCherry subcellular localization in primary roots of *pPin1At:Pin1At-GFP pPIN1:PIN1-mCherry* seedlings at 4 DAG by CLSM.** Pin1At-GFP is expressed in the whole stele cells and partially overlapped with PIN1-mCherry. Scale bar, 10 µm.

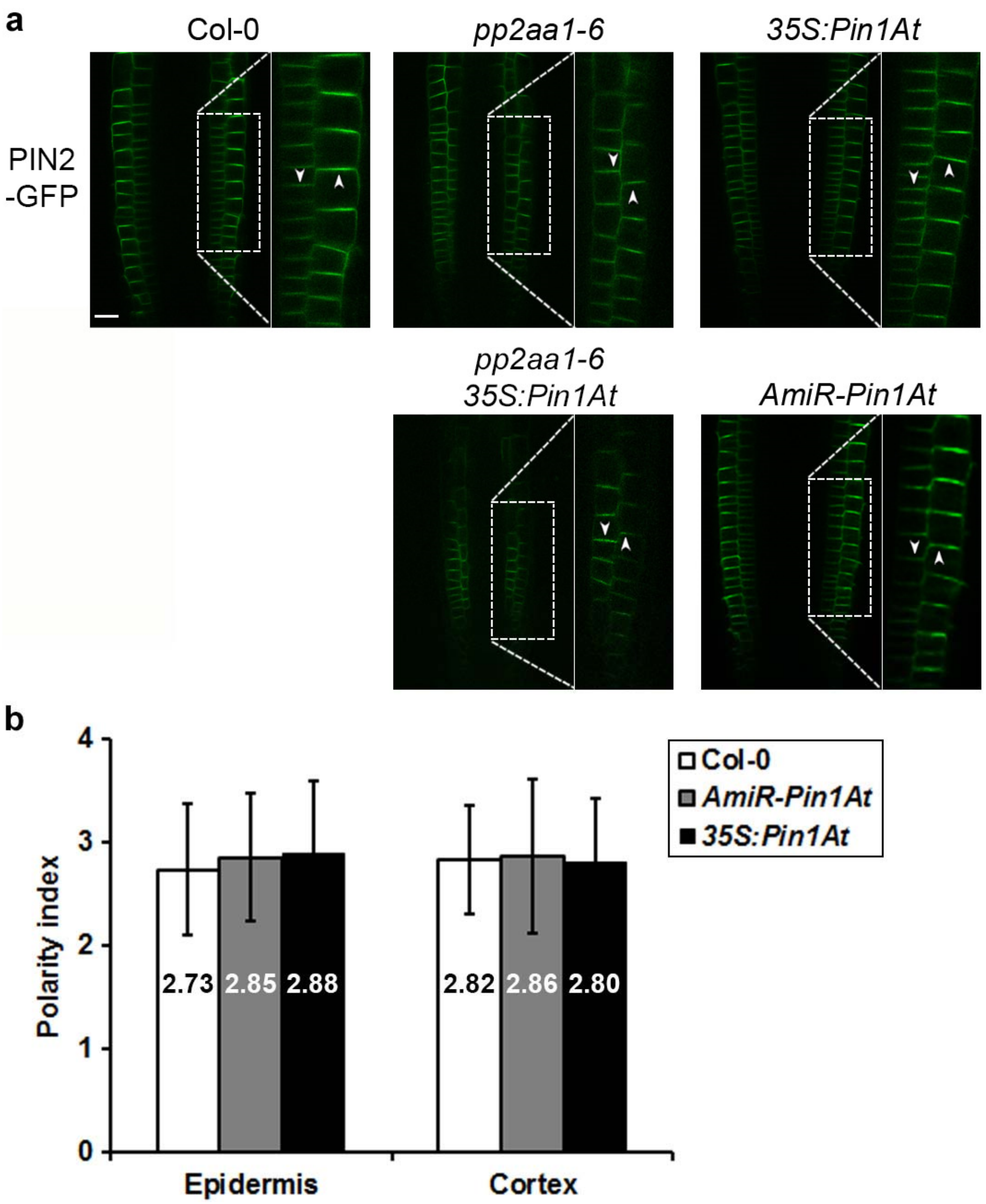


**Supplementary Figure 12. Polar localization of PIN2.** (**a**) Visualization of PIN2-GFP subcellular localization in primary roots of seedlings with different genetic backgrounds at 4 DAG by CLSM. The white dashed boxes in the overview images (left) show the regions of zoomed-in images (right), in which the PIN2-GFP polarity is indicated by arrows. Scale bar, 20 µm. (**b**) Quantification of PIN2-GFP polarity in epidermal and cortical cells in wild-type, *AmiR-Pin1At*, and *35S:Pin1At* primary roots by Leica Application Suite Advanced Fluorescence (LAS AF) software. Polarity index was determined by the ratio of PIN2-GFP intensity at polar versus lateral plasma membrane. The value represents the average ± SD for 40 epidermal or cortical cells from 5 roots for each genotype. There were no statistical differences in polarity index in epidermal and cortical cells between wild-type and *AmiR-Pin1At* or *35S:Pin1At* plants.

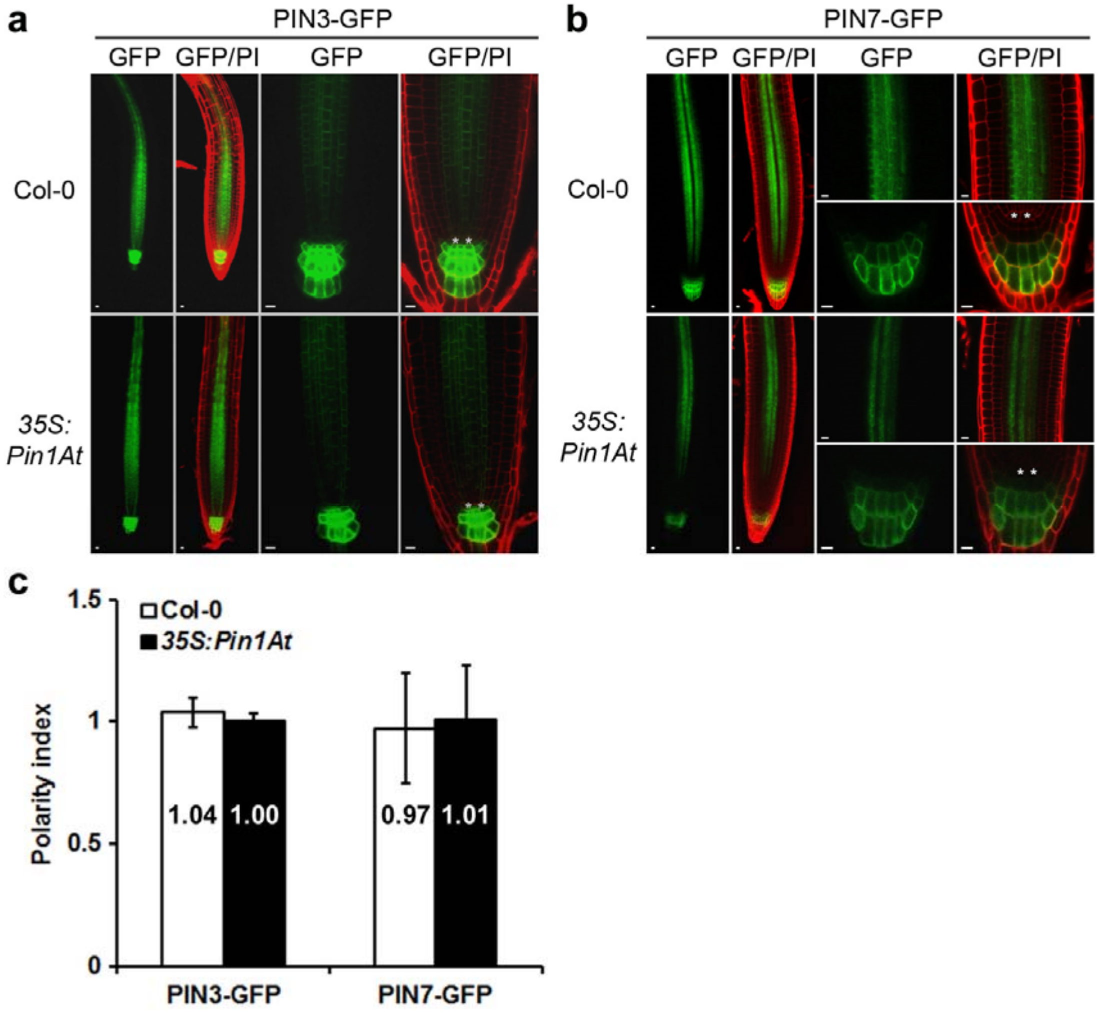


**Supplementary Figure 13. Effect of *35S:Pin1At* on subcellular localization of PIN3 and PIN7 in roots.** Visualization of PIN3-GFP (**a**) and PIN7-GFP (**b**) subcellular localization in primary roots of *pPIN3:PIN3-GFP* and *pPIN7:PIN7-GFP* seedlings, respectively, in either wild-type or *35S:Pin1At* background, at 4 DAG by CLSM. Two asterisks (**) denote quiescent center cells. PI, propidium iodide. Scale bars, 10 µm. (**c**) Quantification of the polarity of PIN3-GFP and PIN7-GFP in columella cells in wild-type and *35S:Pin1At* primary roots by Leica Application Suite Advanced Fluorescence (LAS AF) software. Polarity index was determined by the ratio of the fluorescent intensity at polar versus lateral plasma membrane. The value represents the average ± SD for 40 columella cells from 5 roots for each genotype. There were no statistical differences in polarity index for PIN3-GFP and PIN7-GFP between wild-type and *35S:Pin1At* plants.

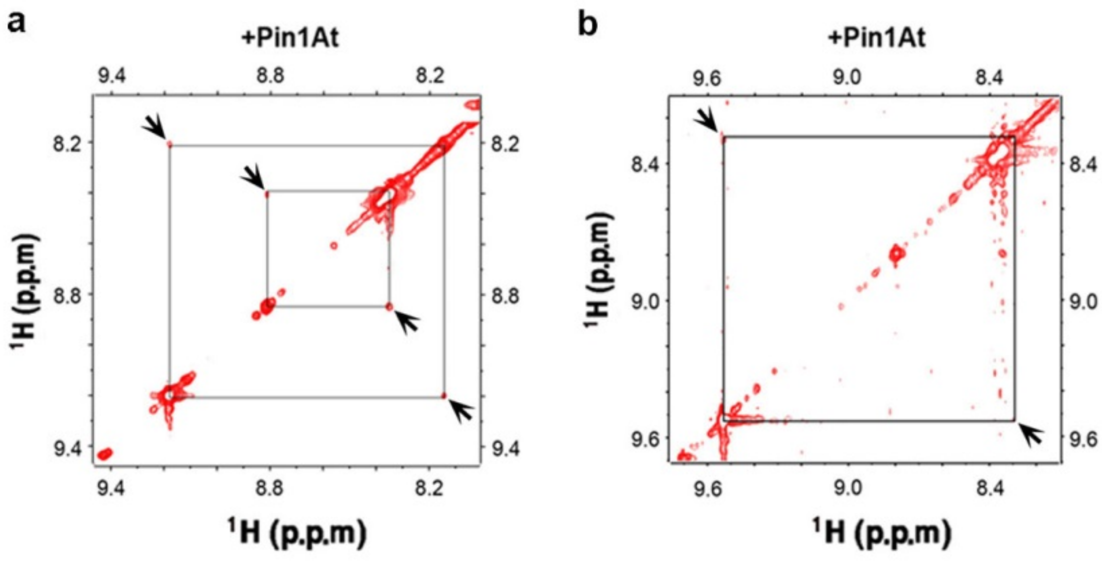


**Supplementary Figure 14. Pin1At catalyzes *cis/trans* isomerization of the peptides corresponding to motifs 2 and 3 in the central PIN1 hydrophilic loop.** (**a,b**) Selected regions of two-dimensional ROESY spectra of the phosphorylated PIN1 peptides in the presence of Pin1At at a mixing time of 110 ms. Exchange cross peaks resulting from Pin1At-catalyzed *cis/trans* isomerization of pThr-Pro motifs are indicated by arrows. The NMR results for the peptides corresponding to motif 2 (**a**) and motif 3 (**b**) are shown.

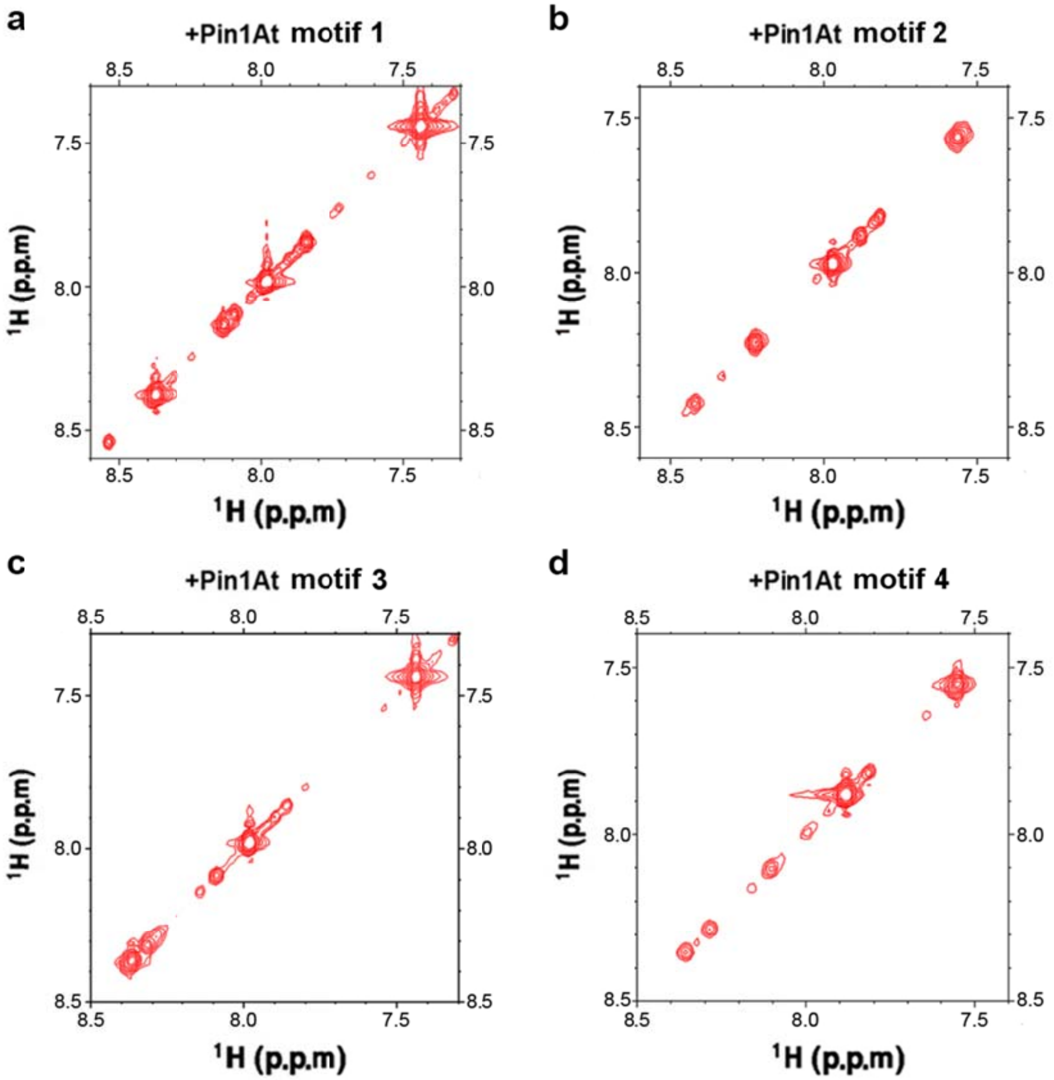


**Supplementary Figure 15. Pin1At does not catalyze the *cis/trans* isomerization of the non-phosphorylated Ser/Thr-Pro motifs in the central PIN1 hydrophilic loop.** Selected regions of two dimensional ROESY spectra of the non-phosphorylated PIN1 peptides corresponding to motifs 1-4 (**a**-**d**) in the presence of Pin1At at a mixing time of 110 ms. There are no exchange cross peaks resulting from Pin1At-catalyzed *cis/trans* isomerization of Ser/Thr-Pro motifs observed in these spectra. Similar negative results are observed in the absence of Pin1At.

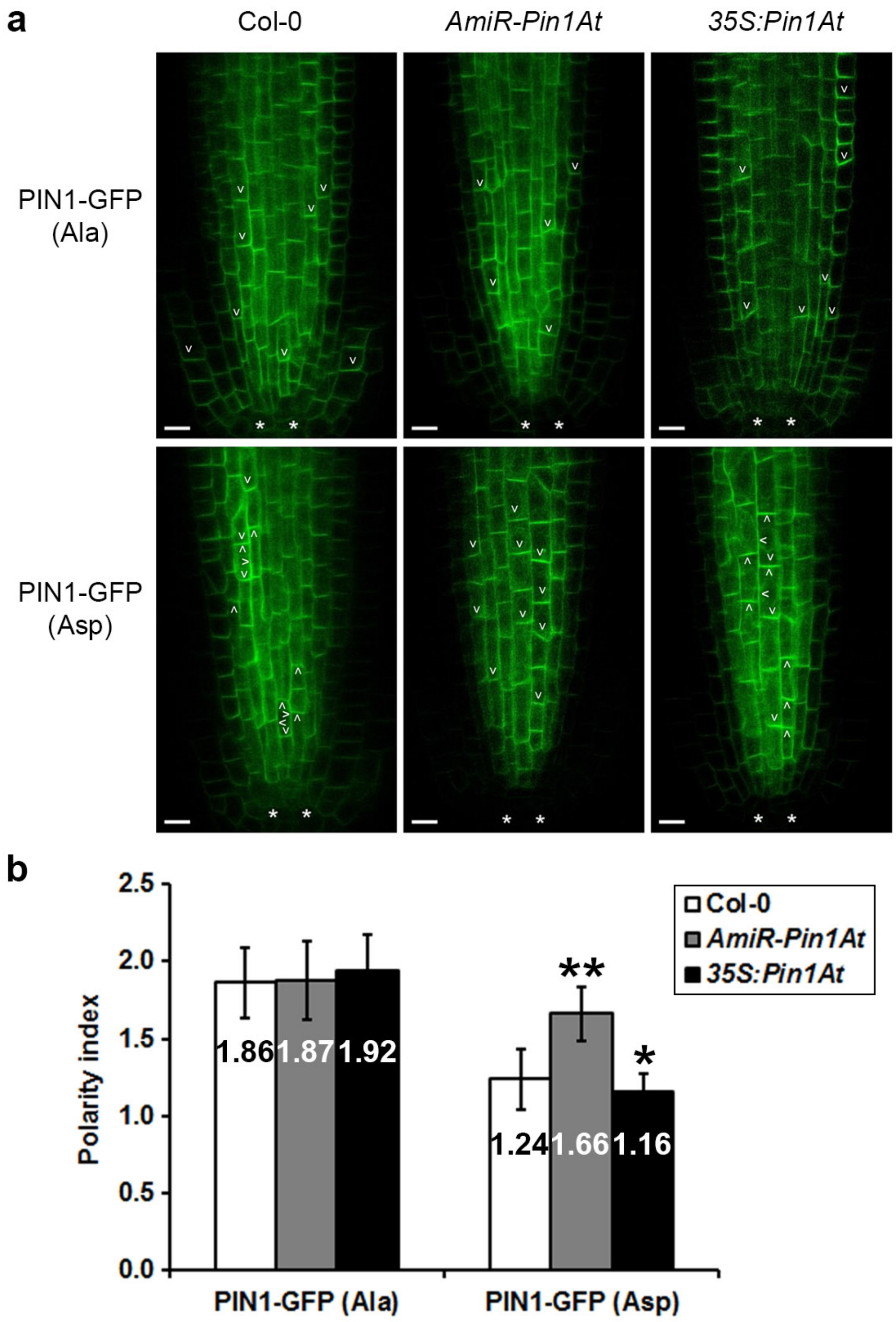


**Supplementary Figure 16. Pin1At effect on PIN1 subcellular localization is mediated by PIN1 phosphorylation at Ser337/Thr340.** (**a**) Visualization of subcellular localization of PIN1-GFP (Ala) (phosphorylation inactivated version, upper panels) and PIN1-GFP (Asp) (phosphomimic version, lower panels) in primary roots of wild-type, *AmiR-Pin1At* and *35S:Pin1At* seedlings at 4 DAG by CLSM. The PIN1-GFP polarity is

indicated by arrows. Two asterisks (**) denote quiescent center (QC) cells. Scale bars, 10 µm. (**b**) Quantification of PIN1-GFP (Ala) and PIN1-GFP (Asp) polarity in stele cells of wild-type, *AmiR-Pin1At*, and *35S:Pin1At* primary roots by Leica Application Suite Advanced Fluorescence (LAS AF) software. Polarity index was determined by the ratio of the fluorescent intensity at polar versus lateral plasma membrane. The value represents the average ± SD for 40 stele cells from 5 roots for each genotype. There were no statistical differences in polarity index for PIN1-GFP (Ala) in wild-type, *AmiR-Pin1At*, and *35S:Pin1At* plants. Asterisks indicate statistically significant differences in polarity index for PIN1-GFP (Asp) in *AmiR-Pin1At* and *35S:Pin1At* compared to that in wild-type plants (two-tailed paired Student's *t* test, *$P < 0.05$, **$P < 0.01$).

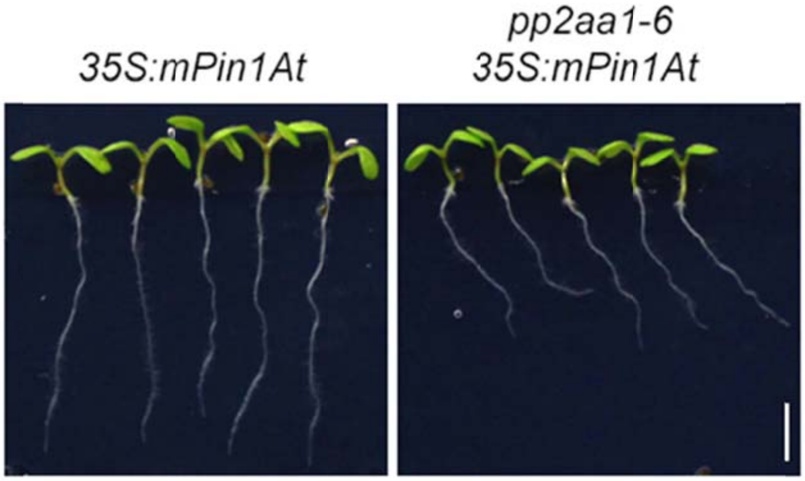


**Supplementary Figure 17. Deletion of the four amino acids in the PPIase domain of Pin1At abolishes the effect of Pin1At on root gravitropism.** Root gravitropic responses of *35S:mPin1At* in wild-type and *pp2aa1-6* plants at 4 DAG are shown. Scale bar, 2.5 mm.

**Supplementary Table 1. Primers used for plasmid construction.**

| Primer Name | Sequence |
|---|---|
| Pin1At-F | 5’-GGAATTCTATATATATGATATACTTTG-3’ |
| Pin1At-P1-R | 5’-ACCCGGGCTTGCTCTTCTTTCGATGCT-3’ |
| Pin1At-P2-R | 5'-ACCCGGGAGCTGTTCTCTTAATGATGT-3' |
| Pin1At-P3-R | 5'-ACCCGGGAATGAGAAATTGAGTAAAAAG-3' |
| Pin1At-GUS-F | 5'-ACATCATTAAGAGAACAGCTCAGTCCCTTATGTTACGTCC-3’ |
| Pin1At-GUS-R | 5’-TTCTATCAGATCAGATAAGGTCATTGTTTGCCTCCCTGCTG-3’ |
| Pin1At-GFP-F | 5’-ACATCATTAAGAGAACAGCTGGAAGTAAAGGAGAAGAACTTTTC-3’ |
| Pin1At-GFP-R | 5’-TTCTATCAGATCAGATAAGGTCATTTGTATAGTTCATCCATGCC-3’ |
| AmiR-Pin1At-I | 5’-GATTAATGATGTGGACTCGACTGTCTCTCTTTTGTATTCC-3’ |
| AmiR-Pin1At-II | 5’-GACAGTCGAGTCCACATCATTAATCAAAGAGAATCAATGA-3’ |
| AmiR-Pin1At-III | 5’-GACAATCGAGTCCACTTCATTATTCACAGGTCGTGATATG-3’ |
| AmiR-Pin1At-IV | 5’-GAATAATGAAGTGGACTCGATTGTCTACATATATATTCCT-3’ |

**Supplementary Table 2. Primer pairs used for quantitative real-time PCR.**

| Primer Name | Sequence |
|---|---|
| TUB2-F | 5’-GAGAATGCTGATGAGTGCATGG-3’ |
| TUB2-R | 5’-AGAGTTGAGTTGACCAGGGAACC-3’ |
| Pin1At-F | 5'-GAAGAGCAAGATGGCGTCGAGAGAC-3' |
| Pin1At-R | 5'-CTGCTCAACGGCGGCTTCTCTAG-3' |
